# Exact solution approaches for the discrete *α*‐neighbor *p*‐center problem

**DOI:** 10.1002/net.22162

**Published:** 2023-06-13

**Authors:** Elisabeth Gaar, Markus Sinnl

**Affiliations:** ^1^ Institute of Production and Logistics Management Johannes Kepler University Linz Linz Austria; ^2^ JKU Business School Johannes Kepler University Linz Linz Austria

**Keywords:** integer programming formulation, location science, min‐max objective, p‐center problem

## Abstract

The discrete α‐neighbor p‐center problem (d‐α‐pCP) is an emerging variant of the classical p‐center problem which recently got attention in literature. In this problem, we are given a discrete set of points and we need to locate p facilities on these points in such a way that the maximum distance between each point where no facility is located and its α‐closest facility is minimized. The only existing algorithms in literature for solving the d‐α‐pCP are approximation algorithms and two recently proposed heuristics. In this work, we present two integer programming formulations for the d‐α‐pCP, together with lifting of inequalities, valid inequalities, inequalities that do not change the optimal objective function value and variable fixing procedures. We provide theoretical results on the strength of the formulations and convergence results for the lower bounds obtained after applying the lifting procedures or the variable fixing procedures in an iterative fashion. Based on our formulations and theoretical results, we develop branch‐and‐cut (B&C ) algorithms, which are further enhanced with a starting heuristic and a primal heuristic. We evaluate the effectiveness of our B&C algorithms using instances from literature. Our algorithms are able to solve 116 out of 194 instances from literature to proven optimality, with a runtime of under a minute for most of them. By doing so, we also provide improved solution values for 116 instances.

## 1 INTRODUCTION

The α‐*neighbor* p‐*center problem* (α‐pCP), proposed by Krumke [25], is an emerging variant of the classical p‐center problem (pCP ) [18] which recently got attention in literature  [6, 10, 31]. In this problem, we are given a set of points and we need to locate p facilities. The goal is to locate the facilities in such a way that the maximum distance between each point and its α‐closest facility is minimized. We note that both a continuous and discrete version of the α‐pCP exist. In the continuous version, the facilities can be located anywhere on the plane, while in the discrete version the given points are also the potential facility locations. In the discrete version all the points where a facility gets located are not considered in the objective function. The α‐pCP can be seen as a robust variant of the pCP , where customers do not need to go to their closest facility, but also have additional α−1 facilities nearby. Thus, the α‐pCP can be a useful modeling approach for applications which are traditionally modeled as pCP , such as emergency service locations and relief actions in humanitarian crisis  [5, 22, 27], where robust solutions are highly relevant.

A formal definition of the discrete α‐pCP (d‐α‐pCP) is as follows  [25, 29, 31]: We are given a set of points N, a positive integer p<|N|, and a positive integer α≤p. For each pair of points i,j∈N we are given a distance $d_{ij}\geq 0$. A feasible solution consists of a subset P⊆N of |P|=p facilities, indicating which facilities are opened. Given a feasible solution P, that is, a set of open facilities P, the set of demand points is defined as N∖P, that is, the set of demand points depends on the chosen feasible solution and consists of all points that are not opened. The α‐distance $d_{\alpha }(P,i)$ for a feasible solution P and a given demand point i∈N∖P is defined as

$$d_{\alpha }(P,i)=\underset{A\subseteq P,|A|=\alpha }{\min}\underset{j\in A}{\max}\{d_{ij}\},$$

so the α‐distance $d_{\alpha }(P,i)$ gives the distance of i to the α‐nearest open facility for the feasible solution P. The objective function value $f_{\alpha }(P)$ of a feasible solution P is defined as

$$f_{\alpha }(P)=\underset{i\in N\setminus P}{\max}d_{\alpha }(P,i).$$

Using these definitions, the d‐α‐pCP can be formulated as

$$\underset{P\subseteq N,|P|=p}{\min}f_{\alpha }(P).$$

We note that the discrete pCP (d‐pCP , also known as *vertex* pCP ) is obtained by setting α=1, if we assume that the distances $d_{ii}=0$ for all i∈N, that is, if we assume that each demand point i is covered if the same facility i is opened. Moreover, instead of assuming that distances between all pairs of points i,j are given, the problem can also be defined on a (non‐complete) graph and the distances are defined as the shortest‐path distances on this graph. With respect to this,  [23] show that the d‐pCP is NP‐hard in general, but there are some classes of graphs such as trees, where the problem can be solved in polynomial time [21].

In this work, we present exact solution approaches for solving the d‐α‐pCP. So far, solution approaches for the α‐pCP focused mostly on the continuous version of the problem. For this version, an iterative exact algorithm based on the connection to a version of the set cover problem is proposed in [10]. We note that for the classical pCP such set cover‐based approaches are well established (for both the continuous and discrete version of the problem), going back to the seminal work of Minieka [28]. Recent set‐cover based approaches for the classical pCP include Chen and Chen [9]; Contardo et al. [11].

In [6] such a set cover‐based approach is used for the continuous version of both the pCP and the α‐pCP. For the d‐α‐pCP the only existing solution approaches in literature are approximation algorithms  [8, 24, 25] and heuristics  [29, 31]. More details on these approaches and on the pCP and other related problems are given in Section 1.2.

### 1.1 Contribution and outline

In this work, we present two different integer programming formulations for the d‐α‐pCP. We also present valid inequalities, (iterative) lifting procedures for some of the inequalities, inequalities that do not change the optimal objective function value and (iterative) variable fixing procedures. We denote the inequalities that do not change the optimal objective function value as *optimality‐preserving* inequalities. The lifting procedures are based on lower bounds to the problem and can be viewed as extension of previous results for the d‐pCP in Gaar and Sinnl [16]. We show that the lower bounds converge to a certain fractional set cover solution when applying the lifting procedure or the variable fixing procedure in an iterative fashion. We also show that we can obtain the optimal objective function value of the semi‐relaxation (in this semi‐relaxation, one set of binary variables of our formulation is kept binary and the other set of binary variables is relaxed) of our second formulation in polynomial time using iterative variable fixing. This can be seen as an extension of a result obtained by Elloumi et al. [14] for the d‐pCP and a fault‐tolerant version of the pCP . Moreover, we provide polyhedral comparisons between the formulations.

Based on these formulations and our theoretical results, we develop branch‐and‐cut (B&C ) algorithms to solve the d‐α‐pCP. These algorithms also contain a starting heuristic and a primal heuristic. We evaluate the effectiveness of our B&C algorithms using instances also used in Sánchez‐Oro et al. [31] and Mousavi [29]. Our algorithms are able to solve 116 out of 194 instances from literature to proven optimality. We also provide improved solution values for 116 out of these 194 instances. Note that these instances are not all the same as the instances for which we manage to prove optimality, as for some instances, the heuristics from literature already found the optimal solution (but of course could not prove optimality, as they are heuristics).

The article is structured as follows: In the remainder of this section, we discuss previous and related work in more detail. Section 2 presents our first integer programming formulation together with valid inequalities, lifted versions of inequalities, optimality‐preserving inequalities and variable fixings. Section 3 contains the same for our second formulation. In Section 4, we provide a polyhedral comparison of the formulations and convergence results for the lower bounds after applying the lifting procedure or the variable fixing procedure in an iterative fashion. In Section 5 we describe the implementation details of our B&C algorithm, including the starting heuristic and the primal heuristic and separation routines. In Section 6 the computational study is presented. Finally, Section 7 concludes the article.

### 1.2 Previous and related work

The pCP is a fundamental problem in location science, dating back to 1965 [18], which has spawned many variations over the years, see, for example, the book‐chapter by [4].

The seminal work of Minieka [28] presented the first exact approach for the pCP and also created a blueprint of a solution algorithm which over the years many other algorithms for either the pCP or also variants of it including the continuous α‐pCP, used as a starting point. Minieka [28] showed that the question whether there exists a feasible solution to the pCP with a given objective function value can be posed as a certain set cover problem. As a consequence the pCP can be solved by iteratively solving such set cover problems. Over the years, many authors  [1, 7, 9, 11, 17, 19, 20] have expanded on this idea to present algorithms to solve the pCP .

Aside from these set cover‐based approaches, there also exist several integer programming formulations for solving the d‐pCP to proven optimality. The classical textbook formulation of the problem (see e.g., [13]) uses facility opening variables and assignment variables and is known to have a bad linear programming relaxation (see, e.g., [33]). In Elloumi et al. [14] an alternative integer programming formulation is presented and the authors show that there are instances where the linear relaxation bounds are provably better than the bounds obtained by the classical textbook formulation. In Ales and Elloumi [2] a modification of this formulation is presented. Regarding our second formulation, which we present in Section 3, we note that there exists a variant of the d‐α‐pCP, in which every point i must be covered α‐times, even if there is a facility opened at i. This variant is sometimes called *fault‐tolerant* pCP (see, e.g., section 6 of Elloumi et al. [14]), although this name has also been used for other problems in literature, including d‐α‐pCP. In section 6 of Elloumi et al. [14] a formulation for the fault‐tolerant pCP extending their formulation for the d‐pCP is sketched. Our second formulation, can be seen as an adaption of this formulation, taking also into account the modification proposed in Ales and Elloumi [2]. In Elloumi et al. [14] it is also shown that a so‐called semi‐relaxation of their formulation, where one of the two sets of binary variables is relaxed, can be solved in polynomial time. They also briefly discuss such a result for their formulation of the fault‐tolerant pCP . We prove a similar result for our second formulation for the d‐α‐pCP in Section 4.3. In Çalık and Tansel [5] another formulation for the d‐pCP is presented and the authors show that the linear programming relaxation of it has the same strength as the relaxation of the formulation of Elloumi et al. [14].

In Gaar and Sinnl [16] the classical textbook formulation was used as starting point for a projection‐based approach, which projected out the assignment variables to obtain a new integer programming formulation for the d‐pCP . Moreover, an iterative lifting scheme for the inequalities in the new formulation was presented. This lifting scheme is based on the lower bound obtained from solving the linear programming relaxation, in which then the lifted inequalities are included and everything is resolved in an iterative fashion. Gaar and Sinnl [16] showed that this procedure converges and the lower bound at convergence is the same lower bound as the one of the semi‐relaxation considered in Elloumi et al. [14]. Furthermore, Gaar and Sinnl [16] also showed that the solution at convergence solves a certain fractional set cover problem. Our first formulation for the d‐α‐pCP, which we present in Section 2, is based on the classical textbook formulation for the d‐pCP and is also suitable for the ideas of Gaar and Sinnl [16] regarding lifting.

For d‐α‐pCP the only existing algorithms with computational results are the GRASP proposed by Sánchez‐Oro et al. [31] and the local search by Mousavi [29]. Aside from these heuristics, there are also works on approximation algorithms  [8, 24, 25] which do not contain computations. The best possible approximation factor of two is obtained by the algorithms presented in Chaudhuri et al. [8]; Khuller et al. [24] under the condition that the distances fulfill the triangle inequalities. We note that in principle set cover‐based approaches such as the one of Chen and Chen [10] also work for the d‐α‐pCP, but [10] focuses on the continuous α‐pCP and presents no computations for the d‐α‐pCP.

## 2 OUR FIRST FORMULATION

In this section we present our first integer programming formulation for the d‐α‐pCP. First, we describe the formulation in Section 2.1. Then we derive valid inequalities, valid inequalities that are based on lower bounds, and optimality‐preserving inequalities in Section 2.2. Next, we detail conditions which allow to fix some of the variables in the linear relaxation in Section 2.3. Finally, we provide some insight on what happens if we relax one set of binary variables of our formulation in Section 2.4.

### 2.1 Formulation

Our first integer programming formulation of the d‐α‐pCP can be viewed as extension of a classical formulation of the d‐pCP (see, e.g., [12] and  [16]). We refer to this classical formulation of the d‐pCP as (PC1) following the notation of Gaar and Sinnl [16]. The formulation (PC1) as well as any other formulations of the d‐pCP which are mentioned in the remainder of this work can be found in the Appendix.

Let the binary variables $y_{j}$ for all j∈N indicate whether a facility is opened at point j. Let the binary variables $x_{ij}$ for all i,j∈N with i≠j indicate whether the point i is assigned to the open facility j. Let the continuous variables z measure the distance in the objective function. Then the d‐α‐pCP can be formulated as

(1a) $$\begin{array}{lll} \left(\text{APC1}\right) & \min\;z, & \end{array}$$

(1b) $$\begin{array}{ll} & \text{s.t.}\;\underset{j\in N}{\sum }y_{j}=p, \end{array}$$

(1c) $$\begin{array}{ll} & \underset{j\in N\setminus \{i\}}{\sum }x_{ij}=\alpha (1-y_{i})\;\forall i\in N, \end{array}$$

(1d) $$\begin{array}{ll} & x_{ij}\leq y_{j}\;\forall i,j\in N,i\neq j, \end{array}$$

(1e) $$\begin{array}{ll} & d_{ij}x_{ij}\leq z\;\forall i,j\in N,i\neq j, \end{array}$$

(1f) $$\begin{array}{ll} & x_{ij}\in \{0,1\}\;\forall i,j\in N,i\neq j, \end{array}$$

(1g) $$\begin{array}{ll} & y_{j}\in \{0,1\}\;\forall j\in N, \end{array}$$

(1h) $$\begin{array}{ll} & z\in \mathbb{R}. \end{array}$$

The constraints (1b) ensure that exactly p facilities are opened. The constraints (1c) make sure that for each point i∈N, the point is either used for opening a facility, or it is assigned to α other open facilities. The constraints (1d) ensure that if a point i is assigned to a facility at point j, then the facility at point j is opened. The constraints (1e) ensure that z takes at least the value of the distance from i to j if i is assigned to j. Thus, z will take at least the maximum distance for assigning i to α facilities, since constraints (1c) ensure the assignment of i to α facilities in case it is not opened. The objective function (1a) minimizes z, that is, it minimizes the maximum assignment distance. The formulation (APC1) has $O(|N{|}^{2})$ variables and $O(|N{|}^{2})$ constraints.

Note that in the formulation (PC1) for the classical formulation of the d‐pCP , the constraint (1e) is included in an aggregated fashion as $\sum_{j\in N}d_{ij}x_{ij}\leq z$ for all i∈N. Furthermore, in the classical d‐pCP also open facilities are included in the demand points. Thus, in (PC1) the variables $x_{ij}$ are required also for i=j, and the right hand‐side is α and not $\alpha (1-y_{i})$ in (1c).

### 2.2 Strengthening inequalities

Due to the fact that (PC1) is typically considered to have bad linear programming bounds (see, e.g., [33]) for the d‐pCP , it could be expected that also (APC1) has a linear relaxation that provides a poor bound. In fact, we confirmed this in preliminary computations, see also Section 6.2. In Section 4.2 we present some theoretical results on the effect of adding the inequalities described in this section to (APC1).

#### 2.2.1 Valid inequalities

The next theorem presents two sets of valid inequalities for (APC1).

Theorem 1 *The inequalities*

(2a) $$\begin{array}{ll} \underset{j\in N\setminus \{i\}}{\sum }d_{ij}x_{ij} & \leq \alpha z\;\forall i\in N, \end{array}$$

(2b) $$\begin{array}{ll} y_{i}+x_{ij} & \leq 1\;\forall i,j\in N,i\neq j \end{array}$$

*are valid inequalities for the formulation (APC1) for the d‐* α *‐* p *CP, that is, when adding (* *2a* *) and (* *2b* *) to (APC1), the set of feasible solutions does not change*.

Clearly (2a) holds for any feasible solution for (APC1), as in this case $\sum_{j\in N\setminus \{i\}}d_{ij}x_{ij}$ is either zero (in case i is opened) or the sum of the distances of the closest, second‐closest, …,α‐closest facility to point i, which is at most α times the distances of the α‐closest facility measured as z.

Furthermore, it is obvious that (2b) holds for any feasible solution for (APC1), as i cannot be assigned to any point j∈N∖{i} if i is opened.

#### 2.2.2 Valid inequalities based on lower bounds

Given a lower bound on the optimal objective function value of the d‐α‐pCP, the inequalities (1e) and (2a) can be lifted, as we show next. The lifting is based on a similar idea recently proposed in  [16] for the d‐pCP .

Theorem 2 *Let* LB *be a lower bound on the optimal objective function value of the d‐* α *‐* p *CP. Then*

(3a) $$\begin{array}{ll} LBy_{i}+\max\{LB,d_{ij}\}x_{ij} & \leq z\;\forall i,j\in N,i\neq j, \end{array}$$

(3b) $$\begin{array}{ll} \max\{LB,d_{ij}\}x_{ij} & \leq z\;\forall i,j\in N,i\neq j, \end{array}$$

(3c) $$\begin{array}{ll} \alpha LBy_{i}+\underset{j\in N\setminus \{i\}}{\sum }\max\{LB,d_{ij}\}x_{ij} & \leq \alpha z\;\;\forall i\in N \end{array}$$

*are valid inequalities for the formulation (APC1) for the d‐* α *‐* p *CP, that is, when adding (* *3c* *), (* *3a* *), and (* *3b* *) to (APC1), the set of feasible solutions does not change*.

For the inequalities (3a), we note that due to constraints (1c) at most one of $y_{i}$ and $x_{ij}$ can take the value one in any feasible solution of (APC1). Thus, the left hand‐side can be at most $\max\{LB,d_{ij}\}$, which is clearly a valid lower bound for z.

Clearly the inequalities (3b) are just a relaxation of (3a) and therefore also valid. The validity of the inequalities (3c) follows from combining the arguments from the proof of the validity of inequalities (2a) with the proof for the validity of the inequalities (3a).

Theorem 2 allows us to add new valid inequalities to the linear relaxation of (APC1), as soon as we have a lower bound LB. We present an iterative scheme exploiting this fact in Section 4.2, where we also analyze the convergence behavior of this scheme.

#### 2.2.3 Optimality‐preserving inequalities

Next we consider optimality‐preserving inequalities. These inequalities may cut off some feasible solutions of (APC1), but do not change the optimal objective function value. In other words, there exists at least one optimal solution to (APC1) which fulfills all these inequalities.

To present the inequalities, let $S_{ij}=\{j^{\prime }\in N:(d_{ij^{\prime }}<d_{ij})\;\text{or}\;(d_{ij^{\prime }}=d_{ij}\;\text{and}\;j^{\prime }<j)\}$, that is, $S_{ij}$ is the set of points $j^{\prime }$ such that $j^{\prime }$ is closer to i than j, or such that $j^{\prime }$ and j are at the same distance to i and $j^{\prime }$ has a smaller index than j. Thus, for any point i, the sets $S_{ij}$ induce an ordering of all points according to their distance to i and their index. We denote this order with $\sigma_{i}$.

Theorem 3 *The inequalities*

(4a) $$\begin{array}{ll} \underset{j\in N_{\alpha }}{\sum }y_{j}+\underset{j\in N\setminus {⋃}_{j^{\prime }\in N_{\alpha }}(S_{ij^{\prime }}\cup \{i,j^{\prime }\})}{\sum }x_{ij}\leq \alpha \;\forall i\in N,\forall N_{\alpha }\subseteq N,|N_{\alpha }|=\alpha & \end{array}$$

*and, if* UB *is the objective function value of a feasible solution of the d‐* α *‐* p *CP, the inequalities*

(4b) $$\begin{array}{ll} \underset{j\in N\setminus \{i\}:d_{ij}\leq UB}{\sum }y_{j}\geq \alpha (1-y_{i})\;\forall i\in N & \end{array}$$

*are optimality‐preserving inequalities for the formulation (APC1) for the d‐* α *‐* p *CP, that is, when adding (* *4a* *) and (* *4b* *) to (APC1), the optimal objective function value does not change*.

Note that the set $N\setminus {⋃}_{j^{\prime }\in N_{\alpha }}(S_{ij^{\prime }}\cup \{i,j^{\prime }\})$ that appears in (4a) can alternatively be described as the set $\left\{j\in N\setminus \{i\}:(d_{ij}>\max_{j^{\prime }\in N_{\alpha }}\{d_{ij^{\prime }}\})\;\text{or}\;(d_{ij}=\max_{j^{\prime }\in N_{\alpha }}\{d_{ij^{\prime }}\}\;\text{and}\;j>\max\{j^{\prime }\in N_{\alpha }:d_{ij}=d_{ij^{\prime }}\})\right\}$ and is the set of facilities j that are further away to i than the furthest facility in $N_{\alpha }$ according to $\sigma_{i}$.

The inequality (4a) ensures that if a certain number β of facilities, that are at most as far away from i than the furthest facility in $N_{\alpha }$, are opened (and thus, i can be assigned to these β facilities), the point i is assigned at most α−β times to facilities that are further away from i than the furthest facility in $N_{\alpha }$. Clearly this is fulfilled for any optimal solution of (APC1), where every facility i is assigned to those α opened facilities, that are the α closest facilities to i according to $\sigma_{i}$. Thus, adding (4a) to (APC1) does not change the optimal objective function value.

Next consider the inequalities (4b). If $y_{i}$ is one in an optimal solution of (APC1), the inequality (4b) is clearly satisfied and thus it does not cut off any optimal solution. Now suppose $y_{i}$ is zero, so location i is not opened. As we know that a feasible solution with objective function value UB exists, it follows that i must be assigned to α facilities at distance at most UB to location i and these α facilities must be opened. Therefore, also in this case (4b) is fulfilled.

Note that the inequalities (4a) from Theorem 3 force an assignment of any location i to those α opened facilities, that are the α closest opened facilities to i according to $\sigma_{i}$. They do so, even when also other assignments would not change the objective function value. Thus, in a sense (4a) are symmetry breaking constraints that forbid certain similar solutions.

### 2.3 Variable fixing

Next, we present a variable fixing condition which can be utilized whenever a feasible solution to the d‐α‐pCP is known. This fixing of variables cuts off feasible solutions, but it does not cut off any optimal solution, that is, it is optimality‐preserving.

Theorem 4 *Let* UB *be the objective function value of a feasible solution of the d‐* α *‐* p *CP. Then when adding the constraints*

(5a) $$\begin{array}{ll} x_{ij}=0\;\forall i,j\in N,i\neq j,d_{ij}>UB & \end{array}$$

*to (APC1), no optimal solution is cut off*.

Clearly in an optimal solution no point i can be assigned to a point j that is further away than UB, thus (5a) is satisfied for any optimal solution.

### 2.4 Relaxing the assignment variables

We now turn our attention to an interesting aspect of (APC1). The classical formulation (PC1) of the d‐pCP has the following nice property: When relaxing the x‐variables in (PC1), that is, replacing $x_{ij}\in \{0,1\}$ with $0\leq x_{ij}$ for all i,j∈N, then the optimal objective function value does not change. Hence, it is not necessary to force the x‐variables to be binary in order to obtain the optimal objective function value of (PC1). This is for example exploited by Gaar and Sinnl [16]. Interestingly, this is not the case anymore for the d‐α‐pCP. To investigate this in detail, let (APC1 – Rx) be the formulation (APC1) with relaxed x‐variables, that is, (APC1‐Rx) is (APC1) without (1f) and with the constraints $0\leq x_{ij}$ for all i,j∈N with i≠j.

**FIGURE 1:**
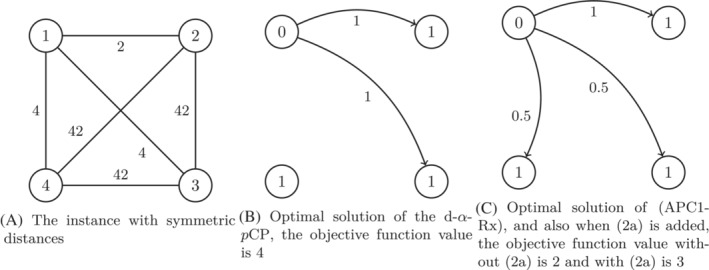
Illustration of Example 5, in which p=3 and α=2. The value in the nodes in (A) is the index of the node and the values near the arcs are the distances. The values in the nodes in (B,C) are the values of the y‐variables in the optimal solution, and the values near the arcs are the values of the x‐variables in the optimal solution. If an arc is not drawn in a solution, this means the corresponding x‐variable takes value zero.

We first consider an example to get some insight. The example is illustrated in Figure 1.

Example 5 Let N={1,2,3,4}, p=3, α=2, $d_{1,2}=2$, $d_{1,3}=d_{1,4}=4$, $d_{2,3}=d_{2,4}=d_{3,4}=42$ and $d_{ij}=d_{ji}$ for all i,j∈N with i≠j.

In this example, it is easy to see that one optimal solution $\left(x^{*},y^{*},z^{*}\right)$ for the formulation (APC1) of the d‐α‐pCP is given as $y_{1}^{*}=0$, $y_{2}^{*}=y_{3}^{*}=y_{4}^{*}=1$, $x_{1,2}^{*}=x_{1,3}^{*}=1$, all other values of $x_{ij}^{*}$ are equal to 0, and $z^{*}=4$. Thus, the optimal objective function value of (APC1) is $z^{*}=4$.

Next consider the solution $\left(x^{\prime },y^{\prime },z^{\prime }\right)$, where $y^{\prime }=y^{*}$, $x_{1,2}^{\prime }=1$, $x_{1,3}^{\prime }=x_{1,4}^{\prime }=0.5$, all other values of $x_{ij}^{\prime }$ are equal to 0, and $z^{\prime }=2$. Clearly, $\left(x^{\prime },y^{\prime },z^{\prime }\right)$ is feasible for (APC1‐Rx) and therefore the optimal objective function value of (APC1‐Rx) is at most 2. Indeed, the optimal objective function value of (APC1‐Rx) is 2 and thus not equal to the optimal objective function value of (APC1).

It is easy to see, that the solution $\left(x^{\prime },y^{\prime },z^{\prime }\right)$ is not feasible anymore for (APC1‐Rx) when the inequalities (2a) are added, as $\left(x^{\prime },y^{\prime },z^{\prime }\right)$ does not fulfill (2a) for i=1. However, the solution $\left(x^{\prime \prime },y^{\prime \prime },z^{\prime \prime }\right)$ with $x^{\prime \prime }=x^{\prime }$, $y^{\prime \prime }=y^{\prime }$ and $z^{\prime \prime }=3$ is feasible. So the optimal objective function value of (APC1‐Rx) with (2a) is at most 3, and indeed it is exactly 3. Thus, it is again not equal to the optimal objective function value of (APC1).

Finally, $\left(x^{\prime \prime },y^{\prime \prime },z^{\prime \prime }\right)$ is not feasible for (APC1‐Rx) with (4a), as $\left(x^{\prime \prime },y^{\prime \prime },z^{\prime \prime }\right)$ does not fulfill the inequality $y_{2}^{\prime \prime }+y_{3}^{\prime \prime }+x_{1,4}^{\prime \prime }\leq \alpha$, which is (4a) for i=1 and $N_{\alpha }=\{2,3\}$. Indeed, the optimal objective function value of (APC1‐Rx) with (4a) coincides with the optimal objective function value of (APC1).

Example 5 shows that (APC1‐Rx) does not necessarily give the same optimal objective function value as (APC1), but there exist instances where after adding (4a), the optimal objective function values coincide. The next result shows that this behavior is not a coincidence.

Theorem 6 (APC1‐Rx) *With (* *4a* *) has the same optimal objective function value as (APC1)*.

Let $\left(x^{*},y^{*},z^{*}\right)$ be an optimal solution of (APC1‐Rx) with (4a). Because of Theorem 3, the optimal objective function value of (APC1) is at least $z^{*}$, so it is enough to show that $z^{*}$ is at least the optimal objective function of (APC1).

To do so, we construct a solution $\left(x^{\circ },y^{\circ },z^{\circ }\right)$ that is feasible for (APC1) with $z^{*}\geq z^{\circ }$. Towards this end consider some i∈N. If $y_{i}^{*}=1$, then let $N_{\alpha }^{i}=\emptyset$. Otherwise, so if $y_{i}^{*}=0$, let $j_{i,k}$ be such that $\sum_{j\in S_{ij_{ik}}\setminus \{i\}}x_{ij}^{*}\leq k-1$ and such that $\sum_{j\in (S_{ij_{ik}}\cup \{j_{ik}\})\setminus \{i\}}x_{ij}^{*}>k-1$ for all k∈{1,2,…,α}. Clearly such $j_{i,k}$ exist because of (1c) and $y_{i}^{*}=0$. Let $N_{\alpha }^{i}=\{j_{i,k}:k\in \{1,\ldots ,\alpha \}\}$. Due to the fact that $x_{ij}^{*}\leq 1$ for all j∈N∖{i}, all $j_{i,k}$ are distinct for different values of k by construction, so $|N_{\alpha }^{i}|=\alpha$. Furthermore,

(6) $$\begin{array}{ll} y_{j}^{*}=1\;\forall j\in N_{\alpha }^{i}, & \end{array}$$

because for such j by construction $x_{ij}^{*}>0$ and $y_{j}^{*}\geq x_{ij}^{*}$ because of (1d). As a consequence, (4a) for $N_{\alpha }=N_{\alpha }^{i}$ implies that $\sum_{j\in N\setminus {⋃}_{j^{\prime }\in N_{\alpha }^{i}}(S_{ij^{\prime }}\cup \{i,j^{\prime }\})}x_{ij}^{*}\leq 0$ and hence $x_{ij}^{*}=0$ for all $j\in N\setminus {⋃}_{j^{\prime }\in N_{\alpha }^{i}}(S_{ij^{\prime }}\cup \{i,j^{\prime }\})$. This, together with (1c) and the fact that $j_{i,\alpha }$ is the facility in $N_{\alpha }^{i}$ furthest away from i according to $\sigma_{i}$, implies that $\sum_{j\in (S_{ij_{i,\alpha }}\cup \{j_{i,\alpha }\})\setminus \{i\}}x_{ij}^{*}=\alpha$. Due to the definition of $j_{i,\alpha }$ this implies that $x_{ij_{i,\alpha }}^{*}=1$. Thus

(7) $$\begin{array}{ll} z^{*}\geq d_{ij_{i,\alpha }}=\underset{j\in N_{\alpha }^{i}}{\max}\{d_{ij}\} & \end{array}$$

because of (1e).

Finally, let $y^{\circ }=y^{*}$, $z^{\circ }=z^{*}$ and let $x_{ij}^{\circ }=1$ if $j\in N_{\alpha }^{i}$ and $x_{ij}^{\circ }=0$ otherwise. Clearly, $x^{\circ }$ and $y^{\circ }$ are binary and $y^{\circ }$ satisfies (1b). Furthermore, by construction of $N_{\alpha }^{i}$, also (1c) and, in particular because of (6), (1d) are satisfied. Furthermore, (1e) is fulfilled because of (7). As a consequence, $\left(x^{\circ },y^{\circ },z^{\circ }\right)$ is feasible for (APC1) with $z^{*}=z^{\circ }$, which finishes the proof.

As a consequence of Theorem 6, relaxing the x‐variables in (APC1) without changing the optimal objective function is possible, whenever the inequalities (4a) are added. Example 5 shows that sometimes these inequalities are indeed necessary to preserve the optimal objective function value.

Note that also additionally including (2a) and (2b) into (APC1‐Rx) with (4a) does not change the optimal objective function value, as these inequalities are valid for (APC1).

## 3 OUR SECOND FORMULATION

In this section we detail our second integer programming formulation of the d‐α‐pCP. First, we present the formulation in Section 3.1. Then we derive a set of valid inequalities in Section 3.2. Finally, we present conditions which allow to fix some of the variables in the linear relaxation in Section 3.3.

### 3.1 Formulation

Our second formulation can be viewed as an extension of the formulation for the d‐pCP proposed by [2], which in turn is a refinement of a formulation of [14] with less constraints and the same linear relaxation bound. We denote the formulation of the d‐pCP by [14] as (PCE) in the same fashion as  [16]. Moreover, we denote the formulation of the d‐pCP by [2] as (PCA). Both (PCE) and (PCA) can be found in the Appendix.

Let $D=\{d_{ij}:i,j\in N,i\neq j\}$ denote the set of all possible distances and let $d_{1}$, …, $d_{K}$ be the values in D, that is, $D=\{d_{1},\ldots ,d_{K}\}$. It is easy to see that the optimal objective function value of the d‐α‐pCP is in D and there are at most (|N|−1)|N| potential optimal values. Furthermore, let $D_{i}=\left({⋃}_{j\in N\setminus \{i\}}\{d_{ij}\}\right)\setminus \{d_{1}\}$, so $D_{i}$ is the set of all distances that are relevant for point i, except for the smallest overall distance.

In this formulation, we have a binary variable $u_{k}$ for each k=2,…,K. This variable indicates whether the optimal objective function value of the d‐α‐pCP is greater than or equal to $d_{k}$, that is, $u_{k}$ is one if and only if the optimal objective function value of the d‐α‐pCP is at least $d_{k}$. Aside from the u‐variables, we also have the binary variables $y_{j}$ for all j∈N to indicate whether a facility is opened at point j similar to the previous formulation. The formulation is denoted as (APC2) and reads as

(8a) $$\begin{array}{llll} (\text{APC2})\; & \min & d_{1}+\overset{K}{\underset{k=2}{\sum }}(d_{k} & -d_{k-1})u_{k}, \end{array}$$

(8b) $$\begin{array}{llll} & \text{s.t.}\;\; & \underset{j\in N}{\sum }y_{j} & =p, \end{array}$$

(8c) $$\begin{array}{lllll} & & u_{k-1} & \geq u_{k} & \forall k\in \{3,\ldots ,K\},\; \end{array}$$

(8d) $$\begin{array}{lllll} & \; & \alpha u_{k}+\underset{j\in N\setminus \{i\}:d_{ij}<d_{k}}{\sum }y_{j} & \geq \alpha (1-y_{i})\;\; & \forall i\in N,\forall d_{k}\in D_{i},\; \end{array}$$

(8e) $$\begin{array}{lllll} & \; & u_{k} & \in \{0,1\}\;\; & \forall k\in \{2,\ldots ,K\},\; \end{array}$$

(8f) $$\begin{array}{lllll} & \; & y_{j} & \in \{0,1\} & \forall j\in N. \end{array}$$

The constraints (8b) ensure that exactly p facilities are opened. The constraints (8c) make sure that if the variable $u_{k}$ is one, indicating that the optimal objective function value is at least $d_{k}$, then also all variables with smaller index are one. These constraints ensure that the objective function (8a) measures the objective function value correctly: in (8a), the coefficient of $u_{k}$ is always the distance‐increment from $d_{k-1}$ to $d_{k}$. Thus, we need that all $u_{k^{\prime }}$ with $k^{\prime }\leq k$ are set to one in order to get a value of $d_{k}$ in the objective function. Finally, constraints (8d) model that for each i∈N, the u‐variables are set in such a way that $u_{k}$ is one, if i is not opened and the α‐nearest open facility to i has distance at least $d_{k}$: In case a facility is opened at point i, that is, $y_{i}$ is one, the constraints are trivially fulfilled. In case no facility is opened at point i, that is, $y_{i}$ is zero, the constraints force $u_{k}$ to be one, or that at least α facilities closer than distance $d_{k}$ to i are opened. The formulation (APC2) has $O(|N{|}^{2})$ variables and $O(|N{|}^{2})$ constraints.

In comparison to the formulation (PCA) for the d‐pCP , we have several modifications in (APC2) for the d‐α‐pCP. First, we have the right hand‐side $1-y_{i}$ instead of just 1 and the sum over all j∈N∖{i} instead of over all j∈N in (8d) as a consequence of the fact that in the d‐α‐pCP opened facilities do not serve as demand points. Furthermore, we have a coefficient α for $u_{k}$ and $1-y_{i}$ in (8d). Finally, we do not include K into the set $D_{i}$, independent from whether there is a facility j with distance $d_{ij}=d_{K}$ or not. This does not influence the correctness of the model, as in the case that there is no facility j with $d_{ij}=d_{K}$ for some i, then for (8d) for i and k=K, the sum $\sum_{j\in N\setminus \{i\}:d_{ij}<d_{k}}y_{j}$ is equal to $p-y_{i}$. This implies that the constraint becomes $\alpha u_{K}\geq \alpha (1-y_{i})-(p-y_{i})$, which is always satisfied because 1≤α≤p holds. Therefore the constraint does not impose a restriction on $u_{K}$, and K can be omitted when defining the set $D_{i}$.

### 3.2 Strengthening inequalities

We have the following valid inequalities.

Theorem 7 *The inequalities*

(9) $$\begin{array}{ll} u_{k}+y_{i}\geq 1\;\forall i\in N,d_{k}\in D_{i},|\{j\in N\setminus \{i\}:d_{ij}<d_{k}\}|<\alpha & \end{array}$$

*are valid inequalities for the formulation (APC2) for the d‐* α *‐* p *CP, that is, when adding (* *9* *) to (APC2), the set of feasible solutions does not change*.

In any optimal solution of (APC2), if a point i is such that it does not have α locations at distance smaller than $d_{k}$, then any feasible solution either has objective function value at least $d_{k}$ (so $u_{k}=1$) or i is opened (so $y_{i}=1$).

We observe the following for the inequalities of Theorem 7.

Observation 8 *For any* i *and* $d_{k}\in D_{i}$ *such that* $|\{j\in N\setminus \{i\}:d_{ij}<d_{k}\}|<\alpha$, *the inequalities (* *8d* *) are dominated by the inequalities (* *9* *), because the former are the latter multiplied by* α *with additional non‐negative terms in the sum on the left hand‐side. Thus, it is not necessary to include (* *8d* *) for any such* i *and* $d_{k}$, *if (* *9* *) is included*.

### 3.3 Variable fixing

Next we present some conditions which allow the fixing of variables. In contrast to (APC1), for which we only have a condition based on an upper bound on the optimal objective function value, for (APC2) we also have a condition which can be utilized with any known lower bound on the optimal objective function value of the d‐α‐pCP.

Theorem 9 *Let* LB *be a lower bound on the optimal objective function value of the d‐* α *‐* p *CP. Then*

(10) $$\begin{array}{ll} u_{k}=1\;\forall k\in \{2,\ldots ,K\},d_{k}\leq LB & \end{array}$$

*are valid equalities for the formulation (APC2) for the d‐* α *‐* p *CP, that is, when adding (* *10* *) to (APC2), the set of feasible solutions does not change*.

Consider any optimal solution for (APC2). If LB is a lower bound on the optimal objective function value of the d‐α‐pCP, then this optimal value is at least $d_{k}$ for any k such that $d_{k}\leq LB$. Therefore, $u_{k}=1$ in this case.

In Section 4.3 we present an iterative scheme for variable fixing based on the optimal solution of the linear programming relaxation of (APC2) which can be seen as extension of Theorem 9.

Theorem 10 *Let* UB *be the objective function value of a feasible solution of the d‐* α *‐* p *CP. Then when adding*

(11) $$\begin{array}{ll} u_{k}=0\;\forall k\in \{2,\ldots ,K\},d_{k}>UB & \end{array}$$

*to (APC2), no optimal solution is cut off*.

Consider any optimal solution for (APC2). If UB is an upper bound on the optimal objective function value of the d‐α‐pCP, then this optimal value is at most $d_{k}$ for any k such that $d_{k}\geq UB$. As a consequence, this optimal value it not greater or equal to any $d_{k}>UB$ and hence $u_{k}=0$ in this case.

## 4 POLYHEDRAL STUDY

In this section we provide a polyhedral study of our two integer programming formulations for the d‐α‐pCP. We start by comparing the basic linear relaxations of the two formulations in Section 4.1. Next, we detail how to obtain the best lower bound based on (APC1), which can be computed in polynomial time, in Section 4.2 and also give a combinatorial interpretation of this best bound. In Section 4.3 we do the same for (APC2). Finally, we compare the two best lower bounds in Section 4.4.

### 4.1 Comparison of basic linear relaxations

Whenever several integer programming formulations of a problem are available, it is an interesting question to compare the corresponding linear relaxations. We note that for the d‐pCP,  [2] proved that the objective function values of the linear relaxations of (PCE) and (PCA) coincide. Furthermore, [14] showed that the objective function value of the linear relaxation of (PCE) is always as least as good as the one of the linear relaxation of (PC1), and they demonstrated that the dominance might be strict by providing an instance where this is the case. Thus, in case of the d‐pCP , both (PCA) and (PCE) dominate (PC1).

**FIGURE 2:**
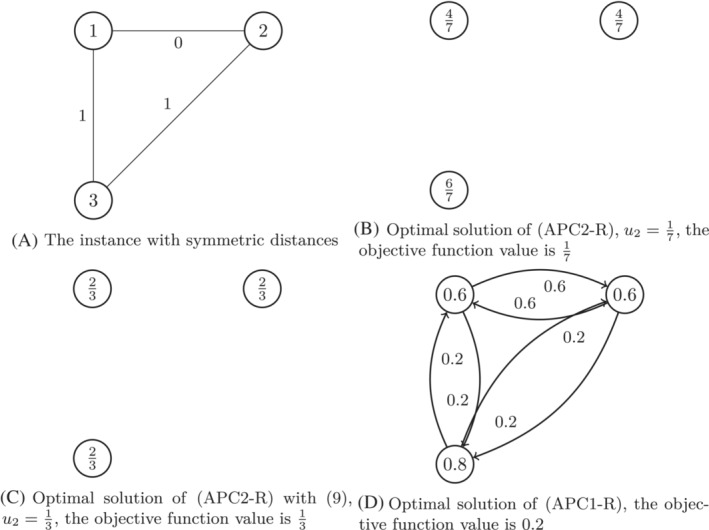
Illustration of Example 11, in which p=2 and α=2. The value in the nodes in (A) is the index of the node and the values near the arcs are the distances. The values in the nodes in (B–D) are the values of the y‐variables in the optimal solution, and the values near the arcs in (D) are the values of the x‐variables in the optimal solution.

**FIGURE 3:**
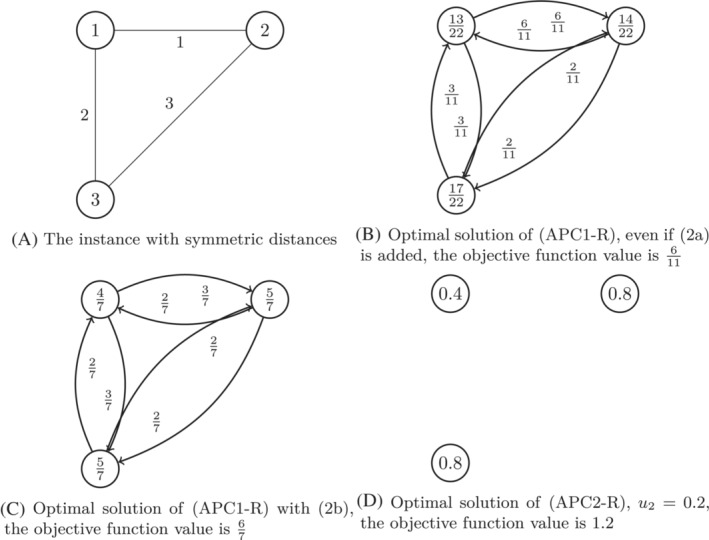
Illustration of Example 12, in which p=2 and α=2. The value in the nodes in (A) is the index of the node and the values near the arcs are the distances. The values in the nodes in (B–D) are the values of the y‐variables in the optimal solution, and the values near the arcs in (B,C) are the values of the x‐variables in the optimal solution.

Let (APC1 – R) be the linear relaxation of (APC1), that is, (APC1‐R) is (APC1) without (1f) and (1g) and with the constraints $0\leq x_{ij}$ for all i,j∈N with i≠j and $0\leq y_{j}\leq 1$ for all j∈N. Let (APC2 – R) be the linear relaxation of (APC2), that is, (APC2‐R) is (APC2) without (8e) and (8f) and with the constraints $0\leq u_{k}\leq 1$ for all k∈{2,…,K} and $0\leq y_{j}\leq 1$ for all j∈N. To study (APC1‐R) and (APC2‐R), we start by considering the following examples, which are illustrated in Figures 2 and 3.

Example 11 Let N={1,2,3}, p=2, α=2, $d_{1,2}=0$, $d_{1,3}=d_{2,3}=1$ and $d_{ij}=d_{ji}$ for all i,j∈N with i≠j.

In the formulation (APC2‐R) we have D={0,1} and $D_{1}=D_{2}=D_{3}=\{1\}$, so K=2. An optimal solution of (APC2‐R) is $y_{1}=y_{2}=\frac{4}{7}$, $y_{3}=\frac{6}{7}$ and $u_{2}=\frac{1}{7}$. Thus, the optimal objective function value of (APC2‐R) is equal to $\frac{1}{7}\approx 0.143$.

This solution is not feasible anymore when adding (9) to (APC2‐R), as $u_{2}+y_{1}=\frac{5}{7}<1$, which violates (9) for i=1 and $d_{k}=1$. An optimal solution of (APC2‐R) with (9) is given as $y_{1}=y_{2}=y_{3}=\frac{2}{3}$ and $u_{2}=\frac{1}{3}$. Therefore, the optimal objective function value of (APC2‐R) with (9) is equal to $\frac{1}{3}\approx 0.333$, which is larger than the optimal objective function value of (APC2‐R).

An optimal solution for (APC1‐R) is $y_{1}=y_{2}=0.6$, $y_{3}=0.8$, $x_{1,2}=x_{2,1}=0.6$, $x_{1,3}=x_{2,3}=x_{3,1}=x_{3,2}=0.2$, and z=0.2, so the optimal objective function value of (APC1‐R) is equal to 0.2. As a consequence, for this instance (APC1‐R) gives a better bound for the d‐α‐pCP than (APC2‐R).

Example 12 Let N={1,2,3}, p=2, α=2, $d_{1,2}=1$, $d_{1,3}=2$, $d_{2,3}=3$ and $d_{ij}=d_{ji}$ for all i,j∈N with i≠j.

For (APC1‐R) an optimal solution is given as $x_{12}=x_{21}=\frac{6}{11}$, $x_{13}=x_{31}=\frac{3}{11}$, $x_{23}=x_{32}=\frac{2}{11}$, $y_{1}=\frac{13}{22}$, $y_{2}=\frac{14}{22}$, $y_{3}=\frac{17}{22}$ and the optimal objective function value of (APC1‐R) is $z=\frac{6}{11}\approx 0.545$. This solution remains feasible when adding (2a), so also for (APC1‐R) with (2a) the optimal objective function value is $z=\frac{6}{11}\approx 0.545$.

However, this solution is not feasible anymore when adding (2b) to (APC1‐R), as $y_{1}+x_{12}=\frac{25}{22}>1$, which violates (2b) for i=1 and j=2. An optimal solution of (APC2‐R) with (2b) is given as $x_{12}=x_{13}=\frac{3}{7}$, $x_{21}=x_{23}=x_{31}=x_{32}=\frac{2}{7}$, $y_{1}=\frac{4}{7}$, $y_{2}=y_{3}=\frac{5}{7}$, and $z=\frac{6}{7}\approx 0.857$. This solution is feasible also when adding (4a). Therefore, the optimal objective function value of (APC1‐R) with (2a) and (2b), and also of (APC1‐R) with (2a), (2b), and (4a) is equal to $\frac{6}{7}\approx 0.857$.

In the formulation (APC2‐R) we have D={1,2,3}, $D_{1}=\{2\}$, $D_{2}=\{3\}$, $D_{3}=\{2,3\}$ and K=3. An optimal solution of (APC2‐R) is $y_{1}=0.4$, $y_{2}=y_{3}=0.8$, $u_{2}=0.2$ and $u_{3}=0$. Thus, the optimal objective function value of (APC2‐R) is equal to 1.2, which is larger than the optimal objective function value of (APC1‐R), even when adding the inequalities (2a), (2b), and (4a) to (APC1‐R). As a consequence, for this instance (APC2‐R) gives a better bound for the d‐α‐pCP than (APC1‐R).

Example 11 shows that the linear relaxation of (APC1) might give strictly better bounds than the linear relaxation of (APC2). In turn, Example 12 shows that the linear relaxation of (APC2) might give strictly better bounds than the linear relaxation of (APC1). Thus, in the case of the d‐α‐pCP the linear relaxations of (APC1) and (APC2) are not comparable.

Furthermore, Example 11 also demonstrates the existence of an instance of the d‐α‐pCP, where including (9) into (APC2‐R) yields a strictly better bound than the one of (APC2‐R). Moreover, Example 12 also shows that there exists an instance where adding (2b) to (APC1‐R) improves the linear relaxation bound.

### 4.2 Best lower bound based on (APC1)

The aim of this section is to derive the best possible bound for (APC1) when utilizing all inequalities derived in Section 2.2. To do so, we investigate Theorem 2 in more detail. In particular, it allows us to add new valid inequalities to the linear relaxation of (APC1), as soon as we have a lower bound LB. Our hope is that including the new valid inequalities for the lower bound LB will give us a new, even better lower bound, with which we can include new, stronger valid inequalities. This leads to an iterative approach to improve the lower bound on the optimal objective function value of the d‐α‐pCP, which is analogous to the approach  [16] have developed for the d‐pCP . They proved that their approach for the d‐pCP converges (i.e., including the valid inequalities for a given lower bound LB does not give a better lower bound, but only LB again) if and only if there is a fractional set cover solution with radius LB that uses at most p sets.

In the following, we investigate a similar iterative approach for the d‐α‐pCP by iteratively adding the inequalities from Theorem 2. Let LB be a lower bound on the optimal objective function value of the d‐α‐pCP and let

(12a) $$\begin{array}{lll} (\text{APCLB})\;{ℒ}_{\alpha }(LB)= & \min z, & \end{array}$$

(12b) $$\begin{array}{ll} & \;\text{s.t.}\;(1b),(1c),(1d), \end{array}$$

(12c) $$\begin{array}{ll} & \;(2b),(3a),(3c), \end{array}$$

(12d) $$\begin{array}{ll} & \;0\leq x_{ij}\;\;\forall i,j\in N,i\neq j, \end{array}$$

(12e) $$\begin{array}{ll} & \;0\leq y_{j}\leq 1\;\forall j\in N, \end{array}$$

(12f) $$\begin{array}{ll} & \;z\geq LB. \end{array}$$

It follows from Theorem 1–3 that ${ℒ}_{\alpha }(LB)$ is again a lower bound on the optimal objective function value of the d‐α‐pCP. Furthermore, it is easy to see that ${ℒ}_{\alpha }(LB)\geq LB$ holds. We now want to establish a condition for the case that adding inequalities from Theorem 2 for a lower bound LB to (APCLB) does not improve the obtained bound ${ℒ}_{\alpha }(LB)$ anymore, that is, a convergence‐condition. It turns out that the following holds.

Theorem 13 *Let* LB *be a lower bound on the optimal objective function value of the d‐* α *‐* p *CP*.

*Then* ${ℒ}_{\alpha }(LB)=LB$ *holds if and only if there is a feasible solution for*

(13a) $$\begin{array}{llll} & \min\; & \underset{j\in N}{\sum }y_{j}, & \end{array}$$

(13b) $$\begin{array}{llll} & \text{s.t.}\;\; & \underset{j\in N\setminus \{i\}:d_{ij}\leq LB}{\sum }y_{j} & \geq \alpha (1-y_{i})\;\;\forall i\in N, \end{array}$$

(13c) $$\begin{array}{llll} & \; & \underset{j\in N\setminus (N_{\beta }\cup \{i\}):d_{ij}\leq LB}{\sum }y_{j} & \geq (\alpha -\beta )(1-y_{i})\;\;\forall i\in N,\forall \beta \in \{1,\ldots ,\alpha \},\forall N_{\beta }\subseteq N\setminus \{i\},|N_{\beta }|=\beta , \end{array}$$

(13d) $$\begin{array}{llll} & \; & 0 & \leq y_{j}\leq 1\;\;\forall j\in N \end{array}$$

*with objective function value at most* p.

We prove each of the two sides of the equivalence in a separate part for the sake of clarity.

**Part 1:** Assume LB is such that ${ℒ}_{\alpha }(LB)=LB$ holds. Let $\left(x^{*},y^{*},z^{*}\right)$ be an optimal solution of (APCLB) in this case, so ${ℒ}_{\alpha }(LB)=z^{*}=LB$. We will finish this part of the proof by showing that $y^{*}$ is a feasible solution for (13) with objective function value at most p.

It is easy to see that (13d) is satisfied because of (12e) and that the objective function value (13a) of $y^{*}$ is p because of (1b).

In order to show that $y^{*}$ fulfills (13b), we can exploit (3c) and get that

$$\alpha LBy_{i}^{*}+\underset{j\in N\setminus \{i\}}{\sum }\max\{LB,d_{ij}\}x_{ij}^{*}\leq \alpha LB\;\forall i\in N$$

holds, which, when splitting the $x_{ij}^{*}$ according to their distance $d_{ij}$, is equivalent to

$$\alpha LBy_{i}^{*}+LB\underset{j\in N\setminus \{i\}:d_{ij}\leq LB}{\sum }x_{ij}^{*}+\underset{j\in N\setminus \{i\}:d_{ij}>LB}{\sum }d_{ij}x_{ij}^{*}\leq \alpha LB\;\forall i\in N.$$

Now we can use (1c) for the first sum with $x_{ij}^{*}$ and obtain

$$\alpha LBy_{i}^{*}+LB\left(\alpha (1-y_{i}^{*})-\underset{j\in N\setminus \{i\}:d_{ij}>LB}{\sum }x_{ij}^{*}\right)+\underset{j\in N\setminus \{i\}:d_{ij}>LB}{\sum }d_{ij}x_{ij}^{*}\leq \alpha LB\;\forall i\in N,$$

which can be simplified to

$$\underset{j\in N\setminus \{i\}:d_{ij}>LB}{\sum }(d_{ij}-LB)x_{ij}^{*}\leq 0\;\forall i\in N.$$

On the left hand‐side this is a sum of non‐negative terms, because $x_{ij}^{*}\geq 0$ due to (12d) and for each term in the sum $(d_{ij}-LB)>0$ holds. Thus, the only way that this can be satisfied is that $x_{ij}^{*}=0$ for all j∈N∖{i} such that $d_{ij}>LB$. This, together with (1c) and (1d) implies that

(14) $$\begin{array}{ll} \alpha (1-y_{i}^{*})=\underset{j\in N\setminus \{i\}}{\sum }x_{ij}^{*}=\underset{j\in N\setminus \{i\}:d_{ij}\leq LB}{\sum }x_{ij}^{*}\leq \underset{j\in N\setminus \{i\}:d_{ij}\leq LB}{\sum }y_{j}^{*}\;\forall i\in N, & \end{array}$$

so $y^{*}$ fulfills (13b).

What is left to show is that $y^{*}$ satisfies (13c). Towards this end, we can use (1d), (14), and (2b) to obtain

$$\begin{array}{llll} \underset{j\in N\setminus (N_{\beta }\cup \{i\}):d_{ij}\leq LB}{\sum }y_{j}^{*} & \geq \underset{j\in N\setminus (N_{\beta }\cup \{i\}):d_{ij}\leq LB}{\sum }x_{ij}^{*}\geq \underset{j\in N\setminus \{i\}:d_{ij}\leq LB}{\sum }x_{ij}^{*}-\underset{j\in N_{\beta }}{\sum }x_{ij}^{*}\; & & \\ & \geq \alpha (1-y_{i}^{*})-|N_{\beta }|(1-y_{i}^{*})\; & & \\ & =(\alpha -\beta )(1-y_{i}^{*})\;\;\;\forall i\in N,\forall \beta \in \{1,\ldots ,\alpha \},\forall N_{\beta }\subseteq N\setminus \{i\},|N_{\beta }|=\beta ,\; & & \end{array}$$

so $y^{*}$ fulfills (13c). Thus, $y^{*}$ is a feasible solution for (13) with objective function value at most p.

**Part 2:** Assume LB is such that there is a feasible solution $y^{\circ }$ for (13) with objective function value of at most p. We will finish this part of the proof in four steps. In the first step we utilize $y^{\circ }$ to construct $y^{*}$, which is feasible for (13) and has an objective function value p. In the second step we use $y^{*}$ to construct $y^{\circ ,i}$ for each i∈N and show that $y^{\circ ,i}$ has a particular property. In the third step we use $y^{\circ ,i}$ to construct $x^{*}$. In the fourth step we show that $\left(x^{*},y^{*},z^{*}\right)$ with $z^{*}=LB$ is a feasible solution for (APCLB), which implies that ${ℒ}_{\alpha }(LB)=LB$ holds.

We start with the first step by constructing $y^{*}$. Let $p^{\circ }$ be the objective function value (13a) of $y^{\circ }$, so $p^{\circ }=\sum_{j\in N}y_{j}^{\circ }$. It follows that $p^{\circ }\leq p$, as $y^{\circ }$ has objective function value at most p. We now construct $y^{*}$ as $y_{j}^{*}=y_{j}^{\circ }+(1-y_{j}^{\circ })\frac{p-p^{\circ }}{|N|-p^{\circ }}$ for all j∈N. We have that $0\leq \frac{p-p^{\circ }}{|N|-p^{\circ }}<1$ because $p^{\circ }\leq p$ and p<|N|. As a consequence, $y_{j}^{*}$ fulfills $0\leq y_{j}^{\circ }\leq y_{j}^{*}\leq 1$ for all j∈N because $y^{\circ }$ fulfills (13d). Furthermore, it holds that

$$\begin{array}{ll} \underset{j\in N}{\sum }y_{j}^{*}=\underset{j\in N}{\sum }\left(y_{j}^{\circ }+(1-y_{j}^{\circ })\frac{p-p^{\circ }}{|N|-p^{\circ }}\right)=\underset{j\in N}{\sum }y_{j}^{\circ }+\frac{p-p^{\circ }}{|N|-p^{\circ }}\underset{j\in N}{\sum }(1-y_{j}^{\circ })=p^{\circ }+\frac{p-p^{\circ }}{|N|-p^{\circ }}(|N|-p^{\circ })=p. & \end{array}$$

Thus, $y^{*}$ is feasible for (13) and has objective function value p.

We proceed with the second step by constructing $y^{\circ ,i}$. For all i∈N we define $y^{\circ ,i}$ in such a way that $y_{j}^{\circ ,i}=\min\{y_{j}^{*},1-y_{i}^{*}\}$ for all j∈N, so in particular $y^{\circ ,i}$ is the component‐wise minimum of $y^{*}$ and $\left(1-y_{i}^{*}\right)$ and $y^{\circ ,i}\leq y^{*}$ holds. We will now show that $y^{\circ ,i}$ fulfills

(15) $$\begin{array}{ll} \underset{j\in N\setminus \{i\}:d_{ij}\leq LB}{\sum }y_{j}^{\circ ,i}\geq \alpha (1-y_{i}^{*})\;\forall i\in N. & \end{array}$$

To do so, let $N_{i}^{\circ }=\{j\in N\setminus \{i\}:d_{ij}\leq LB\;\text{and}\;y_{j}^{\circ ,i}<y_{j}^{*}\}$, so $N_{i}^{\circ }$ is the set of indices j that appear in the sum on the left hand‐side of (15) and fulfill $y_{j}^{*}>1-y_{i}^{*}=y_{j}^{\circ ,i}$.

If $|N_{i}^{\circ }|=0$, then $y_{j}^{\circ ,i}=y_{j}^{*}$ for each term in the sum of (15) and thus (15) is satisfied because $y^{*}$ fulfills (13b).

If $|N_{i}^{\circ }|\geq \alpha$, then $y_{j}^{\circ ,i}=1-y_{i}^{*}$ for all $j\in N_{i}^{\circ }$ implies that

$$\underset{j\in N\setminus \{i\}:d_{ij}\leq LB}{\sum }y_{j}^{\circ ,i}\geq \underset{j\in N_{i}^{\circ }}{\sum }y_{j}^{\circ ,i}=\underset{j\in N_{i}^{\circ }}{\sum }(1-y_{i}^{*})=|N_{i}^{\circ }|(1-y_{i}^{*})\geq \alpha (1-y_{i}^{*}),$$

so also in this case (15) is fulfilled.

If $0<|N_{i}^{\circ }|<\alpha$, then (13c) for $\beta =|N_{i}^{\circ }|$ and $N_{\beta }=N_{i}^{\circ }$ together with the fact that $y_{j}^{\circ ,i}=1-y_{i}^{*}$ for all $j\in N_{\beta }=N_{i}^{\circ }$ shows that

$$\begin{array}{llll} \underset{j\in N\setminus \{i\}:d_{ij}\leq LB}{\sum }y_{j}^{\circ ,i} & =\underset{j\in N\setminus (N_{\beta }\cup \{i\}):d_{ij}\leq LB}{\sum }y_{j}^{\circ ,i}+\underset{j\in N_{\beta }}{\sum }y_{j}^{\circ ,i}\; & & \\ & \geq (\alpha -\beta )(1-y_{i}^{*})+\beta (1-y_{i}^{*})=\alpha (1-y_{i}^{*}),\; & & \end{array}$$

so also in this case (15) is fulfilled. As a consequence, $y^{\circ ,i}$ satisfies (15) in all cases, so for all i∈N.

We continue with the third step, that is, we now construct $x^{*}$. To do so, we first fix a point i∈N. Then let $j_{i}$ be such that $\sum_{j\in S_{ij_{i}}\setminus \{i\}}y_{j}^{\circ ,i}<\alpha (1-y_{i}^{*})$ and such that $\sum_{j\in (S_{ij_{i}}\cup \{j_{i}\})\setminus \{i\}}y_{j}^{\circ ,i}\geq \alpha (1-y_{i}^{*})$. Clearly such a $j_{i}$ exists and $d_{ij_{i}}\leq LB$ because of (15). Then we set $x_{ij}^{*}=y_{j}^{\circ ,i}$ if $j\in S_{ij_{i}}\setminus \{i\}$, we set $x_{ij}^{*}=\alpha (1-y_{i}^{*})-\sum_{j^{\prime }\in S_{ij_{i}}\setminus \{i\}}y_{j^{\prime }}^{\circ ,i}$ if $j=j_{i}$ and we set $x_{ij}^{*}=0$ otherwise. Note that this construction implies that $x_{ij}^{*}=0$ for all j such that $d_{ij}>LB$.

Finally, we are able to do the fourth step, that is, we show that $\left(x^{*},y^{*},z^{*}\right)$ with $z^{*}=LB$ is feasible for (APCLB). By construction, $x_{ij}^{*}\geq 0$, $x_{ij}^{*}\leq y_{j}^{\circ ,i}\leq y_{j}^{*}$ and $x_{ij}^{*}\leq y_{j}^{\circ ,i}\leq 1-y_{i}^{*}$ for all i,j∈N with j≠i, so $\left(x^{*},y^{*},z^{*}\right)$ fulfills (12d), (1d), and (2b). Also $\sum_{j\in N\setminus \{i\}}x_{ij}^{*}=\alpha (1-y_{i}^{*})$ by construction, so (1c) holds. Moreover, by construction $y^{*}$ is a feasible solution of (13) and has objective function value p, so it fulfills (12e) and (1b). Furthermore $z^{*}=LB$, so clearly (12f) is satisfied.

The inequality (3a) is fulfilled if $d_{ij}>LB$, because then $x_{ij}^{*}=0$ and thus $LBy_{i}^{*}\leq LB=z^{*}$ is satisfied as we have already shown that (12e) holds. If $d_{ij}\leq LB$, then the inequality is $LB(y_{i}^{*}+x_{ij}^{*})\leq LB=z^{*}$, which is fulfilled because we already know that (2b) is satisfied. Thus, in any case $\left(x^{*},y^{*},z^{*}\right)$ fulfills (3a).

Finally, we consider (3c). We can utilize $x_{ij}^{*}=0$ whenever $d_{ij}>LB$ and the already shown (1c) to obtain

$$\begin{array}{llll} \alpha LBy_{i}^{*}+\underset{j\in N\setminus \{i\}}{\sum }\max\{LB,d_{ij}\}x_{ij}^{*} & =\alpha LBy_{i}^{*}+LB\underset{j\in N\setminus \{i\}:d_{ij}\leq LB}{\sum }x_{ij}^{*}=\alpha LBy_{i}^{*}+LB\underset{j\in N\setminus \{i\}}{\sum }x_{ij}^{*}\; & & \\ & =\alpha LBy_{i}^{*}+LB\alpha (1-y_{i}^{*})=LB\alpha =\alpha z^{*},\; & & \end{array}$$

so (3c) holds for $\left(x^{*},y^{*},z^{*}\right)$. Therefore, $\left(x^{*},y^{*},z^{*}\right)$ with $z^{*}=LB$ is a feasible solution for (APCLB), which implies that ${ℒ}_{\alpha }(LB)=LB$ holds.

When comparing Theorem 13 to the corresponding result for the d‐pCP , it becomes obvious that (13) is closely related to a fractional set cover problem, where every set has to be covered α times.

We note that the right hand‐side of (13b) is $\alpha (1-y_{i})$, instead of α, which would be the generalization of the result of [16]. This is caused by the fact that i does not need to be covered if it is opened in the d‐α‐pCP, while in the d‐pCP each point needs to be covered. Moreover, the inequalities (13c) are completely new. They make sure that a set cover property is fulfilled not only for all points at most LB away, but also for subsets of these points when removing at most α points. Note that (13b) can be interpreted as (13c) for β=0.

Interestingly, we can pin point which of the inequalities of (APCLB) are responsible for the existence of (13c). To do so, let

(16a) $$\begin{array}{lll} \;{\left(\text{AFSC}\right)}_{\delta }\; & \min\;\underset{j\in N}{\sum }y_{j}, & \end{array}$$

(16b) $$\begin{array}{ll} & \text{s.t.}\;\;\underset{j\in N\setminus \{i\}:d_{ij}\leq \delta }{\sum }\;y_{j}\geq \alpha (1-y_{i})\;\;\forall i\in N, \end{array}$$

(16c) $$\begin{array}{ll} & 0\leq y_{j}\leq 1\;\forall j\in N, \end{array}$$

denote the fraction set cover problem for the d‐α‐pCP for a given δ∈ℝ. Note that ${\left(\text{AFSC}\right)}_{LB}$ is a relaxation of (13). Furthermore, let LB be a lower bound on the optimal objective function value of the d‐α‐pCP and let

$$\begin{array}{ll} \left({\text{APCLB}}^{\prime })\;{ℒ}_{\alpha }^{\prime }(LB\right) & =\min z,\\ & \;\text{s.t.}\;(1b),(1c),(1d),\\ & \;(3b),(3c),\\ & \;(12d),(12e),(12f).\; \end{array}$$

Note that when in (APCLB) the constraint (3a) is relaxed to (3b) and (2b) is removed, then one obtains (APCLB'), so (APCLB') is a relaxation of (APCLB). We are also able to give an interpretation of when the new lower bound ${ℒ}_{\alpha }^{\prime }(LB)$ does not improve the previous lower bound LB in the following theorem.

Theorem 14 *Let* LB *be a lower bound on the optimal objective function value of the d‐* α *‐* p *CP*.

*Then* ${ℒ}_{\alpha }^{\prime }(LB)=LB$ *holds if and only if there is a feasible solution for (AFSC)* 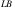 *with objective function value at most* p.

The proof of Theorem 14 is a straight‐forward simplified version of the proof of Theorem 13, where the construction of $y^{\circ ,i}$ in the second part is replaced by using $y^{\circ ,i}=y^{*}$ for all i∈N. Thus, we omit the proof for the sake of brevity.

If we combine the knowledge of Theorem 13 and 14, then we can deduce that the inequalities (2b) and (3a) in (APCLB) (instead of the weaker version (3b) in (APCLB')) are responsible for the existence of (13c) in (13). Thus, the inequalities (13c), which are not present in a straight‐forward generalization of the results of [16] for the d‐pCP to the d‐α‐pCP, are caused by the inequalities (2b) and (3a).

Furthermore, with the help of Theorem 13 and 14 it is easy to see that whenever ${ℒ}_{\alpha }(LB)=LB$ and ${ℒ}_{\alpha }^{\prime }(LB)=LB$ holds for some lower bound LB, then also ${ℒ}_{\alpha }(LB^{\prime })=LB^{\prime }$ and ${ℒ}_{\alpha }^{\prime }(LB^{\prime })=LB^{\prime }$ holds for any $LB^{\prime }>LB$, that is, if the lower bound LB cannot be improved by adding the valid inequalities from Theorem 2, then also no larger lower bound can be improved this way. Thus, it makes sense to define the largest possible lower bounds one can obtain with iteratively adding the valid inequalities from Theorem 2. Let $LB_{\alpha }^{\#}=\min\{LB\in \mathbb{R}:{ℒ}_{\alpha }(LB)=LB\}$ and let ${LB_{\alpha }^{\#}}^{\prime }=\min\{LB\in \mathbb{R}:{ℒ}_{\alpha }^{\prime }(LB)=LB\}$. Our results imply the following relationship.

Corollary 15 *It holds that* $LB_{\alpha }^{\#}\geq {LB_{\alpha }^{\#}}^{\prime }$.

This is a consequence of Theorem 13 and 14.

Next, we point out that both $LB_{\alpha }^{\#}$ and ${LB_{\alpha }^{\#}}^{\prime }$ can be computed efficiently.

Theorem 16 $LB_{\alpha }^{\#}$ *and* ${LB_{\alpha }^{\#}}^{\prime }$ *can be computed in polynomial time*.

A trivial lower bound LB on the optimal objective function value of the d‐α‐pCP is given by $d_{1}$, the smallest element of D. For any given lower bound LB, the computation of ${ℒ}_{\alpha }(LB)$ requires to solve a linear program with a polynomial number of variables and constraints, and thus can be done in polynomial time. Furthermore, there are only a polynomial number of potential values for $LB_{\alpha }^{\#}$, as clearly $LB_{\alpha }^{\#}\in D$ holds, because only for values in D the included variables in the sum in the left hand‐side of (13b) and (13c) change. Thus, whenever we have obtained some new lower bound $LB_{\alpha }^{\#}$, we we know that also $\min_{d_{k}\in D}\{d_{k}\geq LB_{\alpha }^{\#}\}$ is a lower bound. Therefore, it is possible to compute $LB_{\alpha }^{\#}$ in polynomial time.

By same arguments also ${LB_{\alpha }^{\#}}^{\prime }\in D$ and ${LB_{\alpha }^{\#}}^{\prime }$ can be computed in polynomial time.

Thus, not only for the d‐pCP , but also for the d‐α‐pCP the iterative improvement of the lower bound leads to an ultimate lower bound $LB_{\alpha }^{\#}$, which can be computed in polynomial time.

Finally, we want to discuss another interesting aspect about $LB_{\alpha }^{\#}$ and ${LB_{\alpha }^{\#}}^{\prime }$. We have seen in Example 5 that adding the optimality‐preserving inequalities (4a) to a relaxed version of (APC1) improved the bound obtained from the relaxation. Thus, it is a natural question if the bounds $LB_{\alpha }^{\#}$ and ${LB_{\alpha }^{\#}}^{\prime }$ could be further improved by adding (4a) to (APCLB) and (APCLB'), respectively. It turns our that this is not the case.

Theorem 17 *Let* LB *be a lower bound on the optimal objective function value of the d‐* α *‐* p *CP and let* UB *be the objective function value of a feasible solution of the d‐* α *‐* p *CP*. *Let (APCLB*°*) be (APCLB) with (* *4a* *) and (* *4b* *), and denote the optimal objective function value with* ${ℒ}_{\alpha }^{\circ }(LB)$. *Let (APCLB* 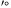 *) be (APCLB') with (* *4a* *) and (* *4b* *), and denote the optimal objective function value with* ${ℒ}_{\alpha }^{\prime \circ }(LB)$.

*Then*

(a)
${ℒ}_{\alpha }^{\circ }(LB)=LB$ *if and only if* ${ℒ}_{\alpha }(LB)=LB$, *and*
(b)
${ℒ}_{\alpha }^{\prime \circ }(LB)=LB$ *if and only if* ${ℒ}_{\alpha }^{\prime }(LB)=LB$.

To prove (a), it is enough to show that ${ℒ}_{\alpha }^{\circ }(LB)=LB$ if and only if there is a feasible solution for (13) with objective function value at most p, because of Theorem 13. To do so, we can follow the proof of Theorem 13. In particular, Part 1 can be used without modifications. Also steps one, two and three of Part 2 can be used without changes. Only in step four we have to additionally show that $\left(x^{*},y^{*},z^{*}\right)$ fulfills (4a) and (4b). Clearly $\left(x^{*},y^{*},z^{*}\right)$ satisfies (4b) due to (13b) and the fact that LB≤UB.

To show that also (4a) is fulfilled we fix some i∈N and some $N_{\alpha }\subseteq N$ with $|N_{\alpha }|=\alpha$. Let $j_{\alpha }\in N_{\alpha }$ be the maximum entry of $N_{\alpha }$ according to $\sigma_{i}$, that is, such that $N_{\alpha }\subseteq (S_{ij_{\alpha }}\cup \{j_{\alpha }\})$. Then (4a) can be reformulated to

(17) $$\begin{array}{ll} \underset{j\in N_{\alpha }}{\sum }y_{j}+\underset{j\in N\setminus (S_{ij_{\alpha }}\cup \{i,j_{\alpha }\})}{\sum }x_{ij}\leq \alpha , & \end{array}$$

so it is enough to show that (17) holds for $\left(x^{*},y^{*},z^{*}\right)$.

If $j_{i}\in (S_{ij_{\alpha }}\cup \{j_{\alpha }\})$, that is, if $j_{i}$ is before $j_{\alpha }$ according to the order $\sigma_{i}$ and thus $j_{i}$ is closer or at the same distance to i than $j_{\alpha }$, then we can deduce that $x_{ij}^{*}=0$ for all $j\in N\setminus (S_{ij_{\alpha }}\cup \{i,j_{\alpha }\})$ by construction, because all of these j are further away from i than $j_{i}$ is. Thus, this implies that (17) is fulfilled in this case, as $|N_{\alpha }|=\alpha$ and $y_{j}^{*}\leq 1$ for all j∈N.

If $j_{i}\notin (S_{ij_{\alpha }}\cup \{j_{\alpha }\})$, that is, if $j_{i}$ is further away to i than $j_{\alpha }$ is, then $x_{ij}^{*}=y_{j}^{\circ ,i}=\min\{y_{j}^{*},1-y_{i}^{*}\}$ holds for all $j\in N_{\alpha }$ by construction. Thus, we can define $\varepsilon_{j}$ such that $y_{j}^{*}=x_{ij}^{*}+\varepsilon_{j}$ for each $j\in N_{\alpha }$, because either $y_{j}^{*}=x_{ij}^{*}$ and $\varepsilon_{j}=0$, or $y_{j}^{*}>x_{ij}^{*}=1-y_{i}^{*}$ and $\varepsilon_{j}=y_{j}^{*}-(1-y_{i}^{*})$. In any case, $0\leq \varepsilon_{j}$ and $\varepsilon_{j}\leq y_{i}^{*}$, as $y_{j}^{*}\leq 1$. This, together with the already shown (1c), implies that

$$\begin{array}{llll} \underset{j\in N_{\alpha }}{\sum }y_{j}^{*}+\underset{j\in N\setminus (S_{ij_{\alpha }}\cup \{i,j_{\alpha }\})}{\sum }x_{ij}^{*} & =\underset{j\in N_{\alpha }}{\sum }(x_{ij}^{*}+\varepsilon_{j})+\underset{j\in N\setminus (S_{ij_{\alpha }}\cup \{i,j_{\alpha }\})}{\sum }x_{ij}^{*}\; & & \\ & \leq \underset{j\in N_{\alpha }}{\sum }\varepsilon_{j}+\underset{j\in N\setminus \{i\}}{\sum }x_{ij}^{*}\leq \alpha y_{i}^{*}+\alpha (1-y_{i}^{*})=\alpha ,\; & & \end{array}$$

so (17) holds also in this case. Thus $\left(x^{*},y^{*},z^{*}\right)$ fulfills (4a), which finishes the proof of (a).

The proof of (b) can be done analogously with the help of Theorem 14 and is therefore skipped.

Theorem 17 shows that adding the optimality‐preserving inequalities (4a) and (4b) to the iterative lifting does not improve the best lower bounds obtained $LB_{\alpha }^{\#}$ and ${LB_{\alpha }^{\#}}^{\prime }$.

### 4.3 Best lower bound based on (APC2)

Next, we analyze (APC2) for the d‐α‐pCP in a similar way [14] and [2] have done with (PCE) and (PCA) for the d‐pCP . To do so, we introduce a *semi‐relaxation* (APC2 – Ry) of (APC2) which is defined as (APC2) with relaxed y‐variables, that is, (APC2‐Ry) is (APC2) without (8f) and with the constraints $0\leq y_{j}\leq 1$ for all j∈N instead. In the same fashion, let (PCE – Ry) be the formulation (PCE) without the constraints $y_{j}\in \{0,1\}$ and with the constraints $0\leq y_{j}\leq 1$ for all j∈N. In case of the d‐pCP , the semi‐relaxation (PCE‐Ry) of  [14] has several interesting properties, which we now investigate in analogous form for the d‐α‐pCP.

#### 4.3.1 Computation in polynomial time

First, for the d‐pCP the optimal objective function value of the semi‐relaxation (PCE‐Ry) can be computed in polynomial time as shown by [14]. Our next aim is to present a procedure for the d‐α‐pCP to compute also the optimal value of the semi‐relaxation (APC2‐Ry) in polynomial time. To do so, we first need the following result.

Lemma 18 *Let* $k^{\prime }$ *be such that*

(18) $$\begin{array}{ll} u_{k}=1\;\forall k\in \{2,\ldots ,K\},k\leq k^{\prime } & \end{array}$$

*are valid equalities for both (APC2) and (APC2‐Ry). Let* $\left(y^{*},u^{*}\right)$ *be an optimal solution of (APC2‐R) with (* *18* *). If* $u^{*}$ *is binary, let* $k^{*}$ *be the largest* k *such that* $u_{k}^{*}=1$. *If* $u^{*}$ *is not binary, let* $k^{*}$ *be the smallest* k *such that* $u_{k}^{*}<1$, *that is*, $u_{k^{*}}^{*}$ *is the first fractional entry of* $u^{*}$. *Then the constraints*

(19) $$\begin{array}{ll} u_{k}=1\;\forall k\in \{2,\ldots ,K\},k\leq k^{*} & \end{array}$$

*are valid equalities for both (APC2) and (APC2‐Ry), that is, when adding (* *19* *) to (APC2) and (APC2‐Ry), the respective sets of feasible solutions do not change*.

If $u^{*}$ is binary, $k^{*}$ is chosen in such a way that the optimal objective function value of (APC2‐R) with (18) is $d_{k^{*}}$. If $u^{*}$ is not binary, then $k^{*}$ is chosen in such a way that the optimal objective function value of (APC2‐R) with (18) is larger than $d_{k^{*}-1}$. Thus, in any case, the optimal objective function value of (APC2‐R) with (18) is larger than $d_{k^{*}-1}$.

Assume that (19) is not a valid equality for (APC2). Then there is a feasible solution $\left(u^{\circ },y^{\circ }\right)$ of (APC2) and there is a $k^{\circ }\leq k^{*}$ such that $u_{k^{\circ }}^{\circ }=0$. Then $u_{k}^{\circ }=0$ for all $k\geq k^{\circ }$ because of (8c) and $u_{k}^{\circ }\leq 1$ for all $k<k^{\circ }$. Thus, the objective function value of $\left(u^{\circ },y^{\circ }\right)$ for (APC2‐R) with (18), which is equal to $d_{1}+\sum_{k=2}^{K}(d_{k}-d_{k-1})u_{k}^{\circ }$, is at most $d_{k^{\circ }-1}$ and therefore it is at most $d_{k^{*}-1}$. Furthermore, $\left(u^{\circ },y^{\circ }\right)$ is feasible for (APC2‐R) with (18), because (APC2‐R) with (18) is a relaxation of (APC2). Thus, the optimal objective function value of (APC2‐R) with (18) is at most $d_{k^{*}-1}$, a contradiction. Therefore, the assumption was wrong and (19) is a valid equality for (APC2).

The fact that (19) is a valid equality for (APC2‐Ry) can be shown analogously.

As a consequence, by applying Lemma 18 in an iterative fashion, we can compute (APC2‐Ry) in polynomial time, as the next results shows.

Theorem 19 *An optimal solution of the semi‐relaxation (APC2‐Ry) can be computed in polynomial time*.

We can compute an optimal solution of (APC2‐Ry) as follows. First, we set $k^{\prime }=1$. Then we solve (APC2‐R) with (18), which is equivalent to (APC2‐R) in the case that $k^{\prime }=1$ holds. Let $\left(y^{*},u^{*}\right)$ be the obtained optimal solution. If $u^{*}$ is binary, it is an optimal solution of (APC2‐Ry). Otherwise, we can apply Lemma 18 to obtain $k^{*}$, update $k^{\prime }=k^{*}$ and solve (APC2‐R) with (18) again. We repeat this, until we obtain a binary $u^{*}$.

Note that $k^{\prime }$ increases at least by one in each iteration, and there are $O(|N{|}^{2})$ many potential values of $k^{\prime }$. Furthermore, in each iteration a linear program with a polynomial number of variables and constraints has to be solved. Thus, this procedure computes an optimal solution of (APC2‐Ry) in polynomial time.

#### 4.3.2 Combinatorial interpretation

A second interesting property of the semi‐relaxation (PCE‐Ry) for the d‐pCP is that  [16] proved that it is connected to the optimal solution of a set cover problem. In particular, the optimal objective function value of (PCE‐Ry) is equal to $d^{*}\in D$ if and only if there is a fractional set cover solution with radius $d^{*}$ that uses at most p sets. It turns out that the following analogous result is also true for (APC2) for the d‐α‐pCP.

Theorem 20 *Let* $d^{*}\in D$. *Then the optimal objective function value of (APC2‐Ry) is equal to* $d^{*}$ *if and only if* $d^{*}$ *is the smallest possible value of* δ *such that there is a feasible solution for* ${\left(\text{AFSC}\right)}_{\delta }$ *with objective function value at most* p.

As (APC2‐Ry) requires the u‐variables to be binary and (8c) has to hold, it is clear that the optimal objective function value of (APC2‐Ry) is a value from D. Furthermore, it is clear that the smallest possible value of δ such that there is a feasible solution for ${\left(\text{AFSC}\right)}_{\delta }$ with objective function value at most p is a value from D, because only for such values the problem ${\left(\text{AFSC}\right)}_{\delta }$ changes. Thus, in order to prove the result it is enough to show that for any δ∈D there is a feasible solution for (APC2‐Ry) with objective function value δ if and only if there is a feasible solution for ${\left(\text{AFSC}\right)}_{\delta }$ with objective function value at most p. We will finish the proof by showing each side of this equivalence in a separate part.

**Part 1:** Let $\left(u^{*},y^{*}\right)$ be a feasible solution of (APC2‐Ry) with objective function value δ∈D. We will finish this part of the proof by showing that $y^{*}$ is a feasible solution for ${\left(\text{AFSC}\right)}_{\delta }$ with objective function value at most p.

Towards this end, let ℓ be such that $\delta =d_{\ell }$. Then (8c) together with (8e) imply that $u_{k}^{*}=1$ for all k∈{1,…,ℓ} and $u_{k}^{*}=0$ for all k∈{ℓ+1,…,K}. Now we fix some i∈N and distinguish two cases.

If there is an element in $D_{i}$ that is larger than $d_{\ell }$, then let $d_{\ell^{\prime }}=\min_{k\in \{1,\ldots ,K\}}\{d_{k}\in D_{i}:d_{k}>d_{\ell }\}$, that is, $d_{\ell^{\prime }}$ is the smallest entry of $D_{i}$ that is larger than $d_{\ell }$. By construction $u_{\ell^{\prime }}^{*}=0$ holds, so (8d) for $d_{k}=d_{\ell^{\prime }}$ implies that

$$\alpha (1-y_{i}^{*})\leq \underset{j\in N\setminus \{i\}:d_{ij}<d_{\ell^{\prime }}}{\sum }y_{j}^{*}=\underset{j\in N\setminus \{i\}:d_{ij}\leq d_{\ell }}{\sum }y_{j}^{*}=\underset{j\in N\setminus \{i\}:d_{ij}\leq \delta }{\sum }y_{j}^{*},$$

so in this case (16b) is satisfied for i.

If there is no element in $D_{i}$ that is larger than $d_{\ell }$, then $d_{ij}\leq d_{\ell }$ for all j∈N∖{i} and with (8b) and 1≤α≤p this implies that

$$\underset{j\in N\setminus \{i\}:d_{ij}\leq \delta }{\sum }y_{j}^{*}=\underset{j\in N\setminus \{i\}:d_{ij}\leq d_{\ell }}{\sum }y_{j}^{*}=\underset{j\in N\setminus \{i\}}{\sum }y_{j}^{*}=p-y_{i}^{*}\geq \alpha (1-y_{i}^{*}),$$

so also in this case (16b) is satisfied for i.

As a result, the inequality (16b) is satisfied by $y^{*}$ in any case. Furthermore, $y^{*}$ fulfills (16c) because it satisfies the relaxation of (8f). The objective function value (16a) of $y^{*}$ is equal to p because of (8b). Thus, $y^{*}$ is feasible for ${\left(\text{AFSC}\right)}_{\delta }$ with objective function value at most p.

**Part 2:** Assume δ∈D is such that there is a feasible solution $y^{\circ }$ for ${\left(\text{AFSC}\right)}_{\delta }$ with objective function value at most p. We will finish this part of the proof by constructing a feasible solution $\left(u^{*},y^{*}\right)$ for (APC2‐Ry) with objective function value δ.

Towards this end, let $u_{1}^{*}=1$ if $d_{k}\leq \delta$ and let $u_{k}^{*}=0$ otherwise. Furthermore, we construct $y^{*}$ from $y^{\circ }$ in the same fashion as in Part 2 of the proof of Theorem 13. In particular, let $p^{\circ }$ be the objective function value (16a) of $y^{\circ }$, so $p^{\circ }=\sum_{j\in N}y_{j}^{\circ }$ and construct $y^{*}$ as $y_{j}^{*}=y_{j}^{\circ }+(1-y_{j}^{\circ })\frac{p-p^{\circ }}{|N|-p^{\circ }}$ for all j∈N. With the same arguments as in the proof of Theorem 13 it follows that $y^{*}$ is feasible for ${\left(\text{AFSC}\right)}_{\delta }$ and has objective function value p.

By construction, $u^{*}$ fulfills (8c) and (8e). Furthermore $y^{*}$ fulfills the relaxation of (8f) because of (16c), and it satisfies (8b) because it has objective function value p for ${\left(\text{AFSC}\right)}_{\delta }$. Next we consider the inequalities (8d) for some i∈N. This inequality is clearly satisfied for any $d_{k}$ such that $u_{k}^{*}=1$. If $d_{k}\in D_{i}$ is such that $u_{k}^{*}=0$, then by construction $d_{k}>\delta$. This together with (16b) implies that

$$\alpha (1-y_{i}^{*})\leq \underset{j\in N\setminus \{i\}:d_{ij}\leq \delta }{\sum }y_{j}^{*}\leq \underset{j\in N\setminus \{i\}:d_{ij}<d_{k}}{\sum }y_{j}^{*}=\underset{j\in N\setminus \{i\}:d_{ij}<d_{k}}{\sum }y_{j}^{*}+\alpha u_{k}^{*},$$

so (8d) holds in any case.

As a consequence, $\left(u^{*},y^{*}\right)$ is feasible for (APC2‐Ry). By construction, and because δ∈D, it follows that the objective function value of $\left(u^{*},y^{*}\right)$ for (APC2‐Ry) is δ, which closes this part of the proof.

### 4.4 Comparison of the best lower bounds

Finally, we compare the best lower bounds obtainable with the two formulations. For the d‐pCP , [16] proved that iteratively using the lower bound information for (PC1) yields a bound, which coincides with the bounds obtained by the semi‐relaxation (PCE‐Ry).

It turns out that this may not the case anymore for the d‐α‐pCP. Towards this end, let $LB_{\alpha }^{*}$ be the optimal objective function of (APC2‐Ry). Then we can deduce the following result.

Theorem 21 *It holds that* $LB_{\alpha }^{\#}\geq {LB_{\alpha }^{\#}}^{\prime }=LB_{\alpha }^{*}$.

This is a consequence of Corollary 15 and Theorem 14 and 20.

As a consequence, for the d‐α‐pCP, when all our valid inequalities are included, the model (APC1) produces as least as good bounds as the semi‐relaxation of (APC2), and might produce better bounds.

## 5 IMPLEMENTATION DETAILS

Since both formulations are of polynomial size, they could be directly given to an integer programming solver for moderately‐sized instances. However, we have implemented B&C approaches based on them which incorporate our valid inequalities and the lifted versions of it, our optimality‐preserving inequalities, a starting heuristic and a primal heuristic, and variable fixing procedures. Moreover, for both formulations, we do not start the solution process with all the inequalities of the formulation, but add some of them on‐the‐fly when needed using separation procedures.

We first describe the starting heuristic and the primal heuristic, which are used by both B&C algorithms in Section 5.1. Then, we give a description of our B&C based on (APC1) in Section 5.2, and a description of our B&C based on (APC2) in Section 5.3. We evaluate the effects of the different ingredients of the B&C algorithms on the performance in Section 6.2.

### 5.1 Starting heuristic and primal heuristic

Our starting heuristic is a greedy heuristic. We initialize the (partial) solution P by randomly picking a location j∈N to open a facility. We then grow P by iteratively adding additional locations to P in a greedy fashion until |P|=p. As a greedy criterion to choose the location to add to P, we take the location j∈N∖P which has the largest α‐distance to |P|. We note that if |P|<α this criterion is not well‐defined, and thus in this case we use the |P|‐distance. We run this heuristic startHeur times before we start with the B&C and initialize the B&C with the best solution found.

Our primal heuristic is a greedy heuristic driven by the values $y^{*}$ of the y‐variables of the linear relaxation at the nodes of the B&C tree. The heuristic simply sorts the locations j∈N in descending order according to $y_{j}^{*}$ and picks the p‐largest ones as a solution. The primal heuristic is implemented within the HeuristicCallback of CPLEX, which is the mixed‐integer programming solver we are using.

### 5.2 Implementation details of the branch‐and‐cut based on (APC1)

#### 5.2.1 Variable fixing

We use the solution value UB from the solution obtained by the starting heuristic to fix the x‐variables to zero as described in Theorem 4 at initialization. During the B&C we continue with this variable fixing procedure by adding these fixings in the UserCutCallback of CPLEX in case an improved primal solution found during the B&C allows additional fixings. This callback gets called by CPLEX whenever the solver encounters a fractional solution during the solution process.

#### 5.2.2 Overall separation scheme

We separate the following inequalities in the branch‐and‐cut, where the order below gives the order in which we do the separation.

1. Valid inequalities (2a)/their lifted version (3c),
2. Inequalities (1e)/their lifted version (3a) from the original formulation,
3. Inequalities (1d) from the original formulation,
4. Optimality‐based inequalities (4b),
5. Optimality‐based inequalities (4a).

The inequalities listed above are separated within the UserCutCallback. Inequalities (1e) and (1d) from the formulation, which are needed for the correctness of our algorithm, are also separated within the LazyConstraintCallback, which gets called by CPLEX for each integer solution (i.e., each potential new feasible solution). We perform at most maxSepRoot separation‐rounds at the root‐node and at most maxSepTree separation‐rounds at the other nodes of the B&C tree. In the root‐node, we add at most maxIneqsRoot violated inequalities in a separation‐round and at the other nodes, we add at most maxIneqsTree violated inequalities. The parameter‐values we used in our computations are given in Section 6. Note that depending on the setting selected, in the computational study not all the inequalities above are actually used. For more details see Section 6.2.

#### 5.2.3 Details about the separation procedures

All inequalities except (4a) are separated by enumeration. We note that the lifted inequalities (3c) and (3a) depend on the current lower bound LB and the inequalities (4b) depend on the current upper bound UB. Thus, these inequalities can potentially be added again in a stronger version for fixed i and j or for a fixed i, when an improved bound becomes available. For this reason, we add them with the CPLEX‐option purgeable, which allows CPLEX to remove added inequalities if they are deemed no longer useful by CPLEX. Moreover, during the B&C tree, we can use the local lower bounds from the nodes of the B&C tree as LB for the inequalities (3a) and (3c). Naturally, the inequalities are then only valid for the subtree starting at this node. CPLEX allows to add such locally valid inequalities with the method addLocal.^1^ When separating the inequalities (1d) and (1e), for each point i we add the ones corresponding to the numInitAPC1 nearest locations j at initialization.

The separation routine for the inequalities (4a) is a heuristic. For a given location i∈N, our goal is to find a set X⊆N∖{i} and a set $N_{\alpha }\subseteq N\setminus (X\cup \{i\})$, with $|N_{\alpha }|=\alpha$ and such that $N_{\alpha }$ contains only j∈N∖(X∪{i}) with $d_{ij}<\min_{j^{\prime }\in X}d_{ij^{\prime }}$. For any such X and $N_{\alpha }$

(20) $$\begin{array}{ll} \underset{j\in N_{\alpha }}{\sum }y_{j}+\underset{j\in X}{\sum }x_{ij}\leq \alpha & \end{array}$$

is a relaxation of the valid inequality (4a), and hence also (20) is a valid inequality. Thus, we want to heuristically find such sets X and $N_{\alpha }$ which maximize $\sum_{j\in N_{\alpha }}y_{j}^{*}+\sum_{j\in X}x_{ij}^{*}$, where $\left(x^{*},y^{*}\right)$ is the solution of the linear programming‐relaxation at the nodes of the B&C tree. Then, if we have that $\sum_{j\in N_{\alpha }}y_{j}^{*}+\sum_{j\in X}x_{ij}^{*}>\alpha$, we have obtained a violated inequality (20) and thus also a violated inequality (4a).

The heuristic proceeds as follows: Let $N_{i}$ be the locations j∈N∖{i} sorted in descending order according to $d_{ij}$. We initialize X with the first entry of $N_{i}$. Based on X, all potential candidates of $N_{\alpha }$ are all j∈N∖(X∪{i}) with $d_{ij}<\min_{j^{\prime }\in X}d_{ij^{\prime }}$. To obtain $N_{\alpha }$, we sort all candidates j according to their $y_{j}^{*}$‐value in descending order, and take the α largest ones. If the inequality (20) implied by X and $N_{\alpha }$ is violated, we stop and add (20) for $N_{\alpha }$ and this X, if not, we continue by adding the next entry from $N_{i}$ to X and repeat the procedure.

#### 5.2.4 Branching priorities

CPLEX allows to set branching priorities on the variables, which it then takes into account during the B&C . We set the priorities of the y‐variables to 100^2^ and the priorities of the x‐variables are left at the default value of zero in order to force CPLEX to branch on the y‐variables first. This is done, as fixing y‐variables is likely to have more structural impact on the linear programming relaxations compared to fixing x‐variables.

### 5.3 Implementation details of the branch‐and‐cut based on (APC2)

#### 5.3.1 Variable fixings

Similar to our approach for (APC1), we use the solution value UB from the solution obtained by the starting heuristic for variable fixing, that is, we fix the u‐variables to zero as described in Theorem 10 at initialization. Moreover, we also continue these fixings in the UserCutCallback whenever an improved incumbent is found.

Furthermore, we also fix the u‐variables to one in the UserCutCallback using the available (local) lower bound LB at the current branch‐and‐cut node and the theory provided in Lemma 18. Note that Lemma 18 allows us to fix one fractional u‐variable in each separation round. Thus, to speed‐up the fixing, we first check if there are k such that $u_{k}$ is fractional and $d_{k}\leq LB$, that is, we check if there are u‐variables that we can fix according to Theorem 9. If yes, under all the u‐variables fulfilling the conditions, we fix the one corresponding to the largest distance. By constraints (8c) this setting will also set all variables corresponding to smaller distances to one. If there is no variable fulfilling this condition, then we use Lemma 18 for fixing. As we use the local lower bound for fixing, we add the fixing with the method addLocal.

#### 5.3.2 Details about the separation scheme

We have implemented a separation routine for the inequalities (8d). This allows us to dynamically add them when needed instead of adding all of them at initialization. This is an attractive option due to the structure of the formulation (in particular constraints (8d)) in combination with Lemma 18. As this lemma provides results to fix u‐variables to one, we may not need to add all inequalities (8d) to correctly measure the objective function value.

Our separation routine is based on enumeration. However, we add at most one violated inequality (8d) per location i∈N in each round of separation. In order to determine which inequality we add, if there is more than one inequality (8d) violated for a location i, we compute $violation(u^{*},y^{*},i,k)=\alpha u_{k}^{*}+\sum_{j\in N\setminus \{i\}:d_{ij}<d_{k}}y_{j}^{*}-\alpha (1-y_{i}^{*})$, where $\left(u^{*},y^{*}\right)$ is the solution of the linear programming‐relaxation at the nodes of the B&C tree. All inequalities with $violation(u^{*},y^{*},i,k)<0$ are violated. Then we calculate the score $s=-violation(u^{*},y^{*},i,k)\cdot d_{ik}$. With the score, we try to find a k which gives a good balance between violation and effect on the objective function value. When we apply the separation‐approach, we initialize our B&C with all the inequalities (8d) corresponding to the nInitAPC2 smallest distances of the instance. Since the inequalities (8d) are needed for correctness of the formulation, we call the separation routine both in the UserCutCallback and also the LazyConstraintCallback.

Regarding the number of separation rounds and the number of added violated inequalities, we use the same strategy as described in Section 5.2.

#### 5.3.3 Branching priorities

Similar to the B&C for (APC1), we set the values of the branching priorities of the y‐variables to 100, and the priorities of the u‐variables are left at the default value of zero.

## 6 COMPUTATIONAL RESULTS

We implemented our B&C algorithms in C++ using CPLEX 20.1. The runs were made on a single core of an Intel Xeon E5‐2670v2 machine with 2.5GHz and 6GB of RAM, and all CPLEX settings were left on their default values, except the branching priorities which we set as described in Section 5. We have set a time limit of 1800 seconds.

### 6.1 Instances

We considered two sets of instances from the literature in our computational study. The details of these sets are given below.

- TSPLIB: This instance set is based on the TSP‐library  [30] and was used in Sánchez‐Oro et al. [31] with α=2,3. In particular, the instances att48, eil101, ch150, pr439, rat575, rat783, pr1002 and rl1323 were used with p∈{10,20,…,130,140}. The number in the instance‐name gives the number of locations |N|. In these instances all locations are given as two‐dimensional coordinates, and the Euclidean distance is used as a distance function. The instance set contains 154 instances.
  We note that Sánchez‐Oro et al. [31] did not use all values of p for all instances. In our computational study we considered the same combinations of instances and p as Sánchez‐Oro et al. [31]. For the used values of p for each instance see for example, Tables 1 and 2.
- pmedian: This instance set is based on the OR‐library  [3]. It was used in Mousavi [29] with α=2. Each instance is given as a graph, and to obtain the distances between all the locations N (nodes in the graph) an all‐pair shortest‐path computation needs to be done. In these instances, all the distances are integer. The number of locations |N| is between 100 and 900, and p is between 5 and 200. Each of these instances has a value of p encoded in the instance. For the concrete values of |N| and p for each instance see Table 5. The instance set contains 40 instances.

**TABLE 1:** Detailed results for instance set TSPLIB with α=2, part one.

			1HSVL				2HVSL				[31]	
Name	|N|	p	UB	LB	t[s]	nBC	UB	LB	t[s]	nBC	UB	t[s]
att48	48	10	**1592.12**	**1592.12**	1.08	1	**1592.12**	**1592.12**	**0.26**	0	**1592.12**	5.14
att48	48	20	**1061.69**	**1061.69**	0.51	0	**1061.69**	**1061.69**	**0.08**	0	1130.85	1.21
att48	48	30	**729.90**	**729.90**	0.34	0	**729.90**	**729.90**	**0.09**	0	936.38	0.42
att48	48	40	**485.06**	**485.06**	0.07	0	**485.06**	**485.06**	**0.02**	0	532.08	0.07
eil101	101	10	**21.21**	**21.21**	30.29	85	**21.21**	**21.21**	**1.79**	13	**21.21**	68.12
eil101	101	20	**13.60**	**13.60**	49.58	576	**13.60**	**13.60**	**2.07**	35	14.14	28.27
eil101	101	30	**11.05**	**11.05**	20.66	364	**11.05**	**11.05**	**1.90**	358	12.00	10.63
eil101	101	40	**9.06**	**9.06**	7.59	50	**9.06**	**9.06**	**0.54**	68	9.43	6.19
eil101	101	50	**8.06**	**8.06**	8.48	340	**8.06**	**8.06**	**0.19**	0	8.60	3.19
eil101	101	60	**7.07**	**7.07**	0.84	0	**7.07**	**7.07**	**0.14**	0	8.25	1.94
eil101	101	70	**6.32**	**6.32**	0.78	0	**6.32**	**6.32**	**0.10**	0	7.28	0.96
eil101	101	80	**5.10**	**5.10**	0.63	0	**5.10**	**5.10**	**0.08**	0	6.32	0.43
eil101	101	90	**4.12**	**4.12**	0.67	1	**4.12**	**4.12**	**0.08**	0	5.00	0.11
eil101	101	100	**2.24**	**2.24**	0.14	0	**2.24**	**2.24**	**0.12**	0	2.83	0.05
ch150	150	10	**205.66**	**205.66**	1276.32	1364	**205.66**	**205.66**	**4.27**	0	**205.66**	223.16
ch150	150	20	**138.69**	**138.69**	465.80	3235	**138.69**	**138.69**	**7.83**	297	141.53	94.75
ch150	150	30	**108.03**	**108.03**	353.45	5441	**108.03**	**108.03**	**22.43**	3614	112.51	55.58
ch150	150	40	**92.67**	**92.67**	226.43	5596	**92.67**	**92.67**	**6.02**	932	96.42	31.74
ch150	150	50	**82.11**	**82.11**	42.61	935	**82.11**	**82.11**	**2.71**	746	87.69	18.10
ch150	150	60	**70.71**	**70.71**	12.19	29	**70.71**	**70.71**	**0.73**	0	78.42	12.24
ch150	150	70	**64.45**	**64.45**	3.90	1	**64.45**	**64.45**	**0.55**	0	68.23	8.20
ch150	150	80	**58.37**	**58.37**	2.80	0	**58.37**	**58.37**	**0.50**	0	64.45	5.57
ch150	150	90	**51.50**	**51.50**	2.08	0	**51.50**	**51.50**	**0.35**	0	62.04	3.63
ch150	150	100	**46.49**	**46.49**	1.48	0	**46.49**	**46.49**	**0.30**	0	53.21	2.35
ch150	150	110	**43.77**	**43.77**	1.29	0	**43.77**	**43.77**	**0.31**	0	51.65	1.36
ch150	150	120	**39.32**	**39.32**	0.98	0	**39.32**	**39.32**	**0.27**	0	50.30	0.72
ch150	150	130	**36.02**	**36.02**	0.52	0	**36.02**	**36.02**	**0.28**	0	46.63	0.31
ch150	150	140	**29.69**	**29.69**	0.48	0	**29.69**	**29.69**	**0.29**	0	42.30	0.14

**TABLE 2:** Detailed results for instance set TSPLIB with α=2, part two.

			1HSVL				2HVSL				[31]	
Name	|N|	p	UB	LB	t[s]	nBC	UB	LB	t[s]	nBC	UB	t[s]
pr439	439	10	4134.01	1944.76	TL	606	**3146.63**	**3146.63**	**12.29**	0	**3146.63**	TL
pr439	439	20	2579.00	1337.89	TL	809	**2177.44**	**2177.44**	**27.75**	136	2226.26	TL
pr439	439	30	1950.00	1020.79	TL	1082	**1475.85**	**1475.85**	**7.73**	0	1500.21	TL
pr439	439	40	1614.00	865.38	TL	1097	**1185.59**	**1185.59**	**12.41**	10	1253.99	TL
pr439	439	50	1308.63	722.83	TL	1182	**984.89**	**984.89**	**9.58**	0	1068.00	1327.63
pr439	439	60	1116.08	653.73	TL	1201	**883.88**	**867.64**	TL	32210	975.00	918.13
pr439	439	70	976.28	596.96	TL	1288	**726.72**	**726.72**	**1050.05**	18676	905.54	639.50
pr439	439	80	855.13	554.50	TL	1629	**637.38**	**637.38**	**178.91**	2513	731.86	509.87
pr439	439	90	742.04	516.31	TL	1121	**583.10**	**575.54**	TL	43141	715.89	405.64
rat575	575	10	160.80	43.39	TL	293	**116.10**	**116.10**	**24.75**	0	116.87	1773.45
rat575	575	20	97.45	37.73	TL	328	**72.62**	**72.62**	**265.55**	1572	74.25	988.02
rat575	575	30	76.03	34.82	TL	433	**59.14**	**56.38**	TL	8426	60.67	666.00
rat575	575	40	64.14	32.93	TL	396	**50.25**	**47.71**	TL	9941	51.40	565.12
rat575	575	50	55.15	31.31	TL	491	**45.88**	**41.79**	TL	9826	46.52	402.00
rat575	575	60	49.25	30.40	TL	468	**41.15**	**37.48**	TL	12053	41.59	290.62
rat575	575	70	44.55	29.24	TL	376	**37.48**	**34.41**	TL	13237	37.70	268.17
rat575	575	80	41.01	28.45	TL	354	**34.99**	**32.02**	TL	19747	35.90	221.25
rat575	575	90	37.59	27.54	TL	357	**32.45**	**29.70**	TL	23787	33.60	158.91
rat575	575	100	36.00	26.68	TL	300	**30.00**	**27.86**	TL	27393	31.38	122.60
rat783	783	10	193.26	41.83	TL	45	**135.25**	**135.25**	**34.92**	0	138.60	TL
rat783	783	20	109.42	38.17	TL	141	**83.10**	**83.10**	**25.68**	0	86.38	TL
rat783	783	30	92.05	34.79	TL	134	**67.88**	**66.21**	TL	2819	70.84	1717.02
rat783	783	40	75.72	32.85	TL	160	**57.43**	**55.60**	TL	4464	60.14	1695.86
rat783	783	50	65.19	31.76	TL	100	55.04	**49.20**	TL	2802	**52.80**	1212.41
rat783	783	60	56.59	31.17	TL	151	49.04	**44.05**	TL	3872	**48.75**	1044.99
rat783	783	70	53.23	30.48	TL	90	**44.20**	**40.31**	TL	4542	44.41	1038.25
rat783	783	80	49.65	29.97	TL	107	**41.68**	**37.36**	TL	5300	42.43	748.93
rat783	783	90	46.39	29.26	TL	99	40.36	**35.00**	TL	6154	**39.20**	722.81
rat783	783	100	42.64	28.76	TL	99	37.64	**32.98**	TL	7309	**37.48**	536.23
pr1002	1002	10	5481.79	1223.80	TL	0	**3853.89**	**3853.89**	**48.07**	0	**3853.89**	TL
pr1002	1002	20	3479.22	1130.48	TL	0	**2593.26**	**2543.32**	TL	3359	2710.17	TL
pr1002	1002	30	2731.30	1009.07	TL	0	**2059.73**	**2008.76**	TL	4002	2150.58	TL
pr1002	1002	40	2214.16	992.40	TL	18	**1746.42**	**1702.84**	TL	7920	1811.77	TL
pr1002	1002	50	1990.60	949.95	TL	3	**1523.15**	**1478.03**	TL	3555	1619.41	TL
pr1002	1002	60	1733.49	914.11	TL	0	**1403.57**	**1315.17**	TL	6229	1431.78	TL
pr1002	1002	70	1555.63	851.41	TL	0	1372.95	**1204.16**	TL	5818	**1346.29**	TL
pr1002	1002	80	1443.09	825.63	TL	3	1253.99	**1104.54**	TL	6891	**1253.00**	TL
pr1002	1002	90	1365.65	803.99	TL	22	**1131.37**	**1033.32**	TL	9010	1170.47	1696.72
pr1002	1002	100	1270.99	777.90	TL	0	**1070.05**	**982.98**	TL	15670	1079.35	1337.80
rl1323	1323	10	6657.78	1138.05	TL	0	**4554.09**	**4554.09**	**660.59**	0	4694.15	TL
rl1323	1323	20	4025.01	1020.45	TL	0	**3055.56**	**3016.84**	TL	252	3227.00	TL
rl1323	1323	30	3209.07	737.33	TL	0	2913.42	**2372.10**	TL	224	**2563.30**	TL
rl1323	1323	40	2592.84	916.48	TL	0	**2039.56**	**1972.47**	TL	482	2166.96	TL
rl1323	1323	50	2248.99	888.87	TL	0	1958.61	**1745.58**	TL	1390	**1907.69**	TL
rl1323	1323	60	2027.10	861.87	TL	0	**1710.60**	**1566.59**	TL	867	1735.40	TL
rl1323	1323	70	1868.99	823.70	TL	0	1647.07	**1415.82**	TL	1425	**1595.20**	TL
rl1323	1323	80	1702.98	802.17	TL	0	1536.00	**1302.31**	TL	1300	**1440.89**	TL
rl1323	1323	90	1576.08	782.66	TL	0	**1329.66**	**1210.04**	TL	1833	1374.72	TL
rl1323	1323	100	1468.95	768.94	TL	0	**1278.10**	**1126.16**	TL	2060	1293.63	TL

### 6.2 Analysis of the ingredients of our branch‐and‐cut algorithms

To analyze the effect of the ingredients of our B&C algorithms, we performed a computational study on a subset of the instances, namely the instances att48, eil101, ch150. We compare the following different settings for the B&C based on (APC1):

- 1: Directly solving (APC1) without any additional ingredients
- 1H: Adding the starting heuristic, the primal heuristic and the variable fixing based on the upper bound according to Theorem 4
- 1HS: Setting 1H with separation of the inequalities (1d) and the inequalities (1e) (instead of adding them in the beginning) as described in Section 5.2
- 1HSV: Setting 1HS together with the valid inequalities (2a) and (2b)
- 1HSVL: Setting 1HSV together with the lifted version (3a) of the inequalities (1e) and also the lifted version (3c) of the inequalities (2a)
- 1HSVLO: Setting 1HSVL together with the optimality‐preserving inequalities (4a) and (4b)

For the B&C based on (APC2) the following settings are considered:

- 2: Directly solving (APC2) without any additional ingredients
- 2H: Adding the starting heuristic, the primal heuristic and the variable fixing based on the upper bound according to Theorem 10
- 2HV: Setting 2H with the valid inequalities (9) replacing the corresponding inequalities (8d) according to Observation 8
- 2HVS: Setting 2HV with separation of the inequalities (8c) (instead of adding them in the beginning) as described in Section 5.3
- 2HVSL: Setting 2HVS with the variable fixing based on the lower bound according to Theorem 9 and Lemma 18 as described in Section 5.3

The following parameter values were used for the B&C algorithms, they were determined in preliminary computations: startHeur: 10, maxIneqsRoot: 50, maxIneqsTree: 20, maxSepRoot: 100, maxSepTree: 1, numInitAPC1: 10, numInitAPC2: 100 for the instance set TSPLIB and 10 for the instance set pmedian. We have used a different parameter for numIntiAPC2 depending on the instance set, as the distance‐structure of the instances is quite different. In particular, for TSPLIB the distances are essentially unique (as they are Euclidean distances) while for pmedian many are similar (as they are shortest path distances on a graph). Thus, for pmedian we would often add all inequalities (8d) at initialization for a parameter value of 100, as there are usually less than 100 different distances in an instance.

**FIGURE 4:**
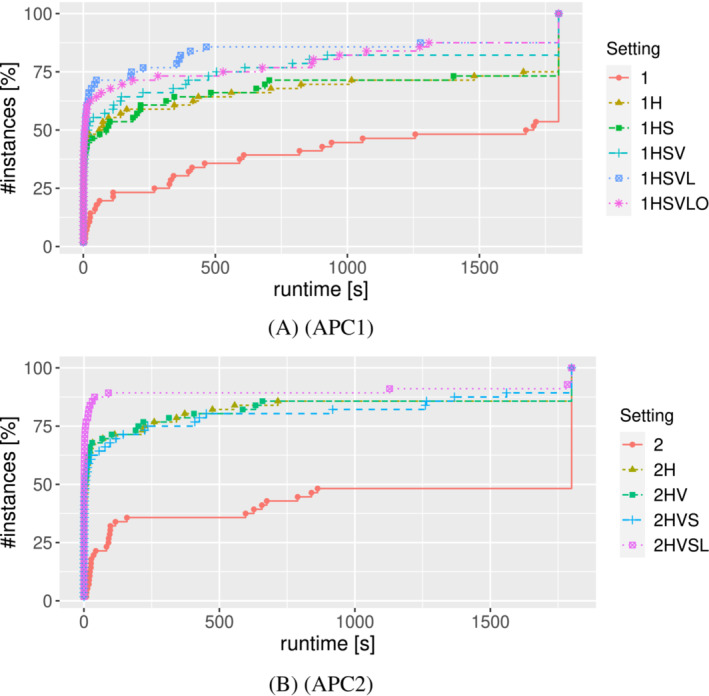
Runtime for different settings of our B&C algorithms on a subset of the instances.

In Figure 4A,B, we show a plot of the runtimes. We see that for both formulations the largest positive effect is achieved by adding the heuristics with the associated variable fixing based on the upper bound. This can be explained by the fact that with the variable fixing the linear programs which are needed to be solved are getting much smaller. Moreover, the lifting procedures for (APC1) and the variable fixing based on the lower bound for (APC2) also have a discernible (incremental) effect. This is in line with both the computational results in Gaar and Sinnl [16] for a similar lifting procedure for the d‐pCP , and the theoretical result provided in Section 4.

Starting to use separation of inequalities which are needed in the formulations (i.e., settings 1HS and 2HVS) instead of adding all of these inequalities at initialization has a rather neutral effect on the selected instances. This can be explained by the fact that these instances are quite small, for the larger instances in our sets, preliminary computations showed that we cannot even solve the root‐relaxation (for both (APC1) and (APC2)) due to either running into the time limit or due to exceeding the available memory.

The valid inequalities (9) also have no visible effect. A potential explanation of this is that modern mixed‐integer programming solvers like CPLEX are quite effective in strengthening given inequalities and may already transform (8c) into (9) automatically whenever it is possible. Finally, adding the optimality‐preserving inequalities for (APC1) has a negative effect. This is consistent with Theorem 17, which shows that at convergence the inequalities (4a) and (4b) are not further improving the bound.

### 6.3 Comparison with approaches from the literature

In this section we provide a detailed comparison with the existing approaches from literature, namely the GRASP of Sánchez‐Oro et al. [31] and the local search of Mousavi [29] on the instances used in the respective works. We compare the existing approaches with the best settings for both of our B&C algorithms, that is, 1HSVL for the one based on (APC1) and 2HVSL for the one based on (APC2).

In Tables 1, 2, 3, 4 we give the comparison with Sánchez‐Oro et al. [31]. For our approaches we report the runtime (columns t[s] with entry TL indicating that the time limit of 1800 seconds was reached), the obtained upper bound (i.e., the objective function value of the best obtained solution, columns UB) and lower bound (columns LB) and the number of nodes in the B&C tree (columns nBC). Since the approach of Sánchez‐Oro et al. [31] is a heuristic, only upper bounds and runtime can be reported for their approach. We note that the runs in Sánchez‐Oro et al. [31] were made on a AMD Ryzen 5 3600 with 2.2 GHz and 16GB RAM. The best values for UB, LB and runtime are indicated in bold in the tables. For the runtime, we just consider our branch‐and‐cut approaches, while for the UB we consider all three approaches to determine these best values.

**TABLE 3:** Detailed results for instance set TSPLIB with α=3, part one.

			1HSVL				2HVSL				[31]	
Name	|N|	p	UB	LB	t[s]	nBC	UB	LB	t[s]	nBC	UB	t[s]
att48	48	10	**2081.57**	**2081.57**	10.41	31	**2081.57**	**2081.57**	**0.89**	103	2186.31	6.72
att48	48	20	**1283.35**	**1283.35**	3.47	14	**1283.35**	**1283.35**	**0.53**	48	1374.48	1.61
att48	48	30	**949.29**	**949.29**	1.53	0	**949.29**	**949.29**	**0.10**	0	1011.66	0.54
att48	48	40	**645.88**	**645.88**	0.13	0	**645.88**	**645.88**	**0.06**	0	675.00	0.08
eil101	101	10	**29.43**	**29.43**	368.66	1338	**29.43**	**29.43**	**15.66**	1092	**29.43**	92.44
eil101	101	20	**17.80**	**17.80**	363.10	3745	**17.80**	**17.80**	**18.45**	2253	18.03	43.73
eil101	101	30	**13.15**	**13.15**	405.18	9499	**13.15**	**13.15**	**14.06**	1567	14.14	19.37
eil101	101	40	**11.18**	**11.18**	179.44	6421	**11.18**	**11.18**	**3.39**	800	12.04	9.71
eil101	101	50	**9.43**	**9.43**	20.20	424	**9.43**	**9.43**	**1.15**	449	10.63	4.74
eil101	101	60	**8.06**	**8.06**	6.01	156	**8.06**	**8.06**	**0.45**	220	9.06	2.22
eil101	101	70	**7.28**	**7.28**	2.28	0	**7.28**	**7.28**	**0.22**	25	8.54	1.06
eil101	101	80	**6.40**	**6.40**	0.84	0	**6.40**	**6.40**	**0.12**	0	7.28	0.44
eil101	101	90	**5.00**	**5.00**	0.43	0	**5.00**	**5.00**	**0.09**	0	6.08	0.11
eil101	101	100	**2.83**	**2.83**	0.14	0	**2.83**	**2.83**	**0.07**	0	**2.83**	0.05
ch150	150	10	**297.96**	205.79	TL	1556	**297.96**	**297.96**	**30.88**	828	298.56	398.03
ch150	150	20	178.21	143.05	TL	3732	**176.47**	**176.47**	**90.44**	4446	179.71	150.94
ch150	150	30	140.06	121.29	TL	11797	**137.46**	**137.46**	**1128.29**	75456	146.41	78.08
ch150	150	40	114.58	108.32	TL	30854	**114.47**	**111.55**	TL	157239	119.22	52.10
ch150	150	50	**100.34**	96.71	TL	38009	100.47	**98.04**	TL	244637	108.03	26.70
ch150	150	60	**90.58**	86.79	TL	55403	**90.58**	**89.09**	TL	379556	97.46	17.78
ch150	150	70	**83.19**	79.80	TL	78518	83.33	**81.91**	TL	475149	92.82	13.10
ch150	150	80	**74.93**	**74.93**	**182.44**	6308	**74.93**	**74.93**	1784.19	621412	83.38	8.34
ch150	150	90	**67.73**	**67.73**	**18.17**	487	**67.73**	**67.73**	39.35	27266	79.81	4.75
ch150	150	100	**63.42**	**63.42**	8.67	19	**63.42**	**63.42**	**1.34**	517	69.35	3.23
ch150	150	110	**59.04**	**59.04**	13.41	424	**59.04**	**59.04**	**2.39**	1753	67.22	1.85
ch150	150	120	**52.97**	**52.97**	2.33	0	**52.97**	**52.97**	**0.55**	0	61.29	0.95
ch150	150	130	**44.46**	**44.46**	1.09	0	**44.46**	**44.46**	**0.33**	0	57.50	0.41
ch150	150	140	**38.56**	**38.56**	0.55	0	**38.56**	**38.56**	**0.30**	0	52.20	0.16

**TABLE 4:** Detailed results for instance set TSPLIB with α=3, part two.

			1HSVL				2HVSL				[31]	
Name	|N|	p	UB	LB	t[s]	nBC	UB	LB	t[s]	nBC	UB	t[s]
pr439	439	10	5997.08	2410.47	TL	582	**4050.31**	**4050.31**	**53.02**	179	4076.23	TL
pr439	439	20	3505.89	1548.18	TL	769	**2683.28**	**2683.28**	**79.62**	786	2726.03	TL
pr439	439	30	2520.17	1209.18	TL	1111	**2065.49**	**2065.49**	**1082.90**	7280	2231.73	TL
pr439	439	40	2102.38	1014.28	TL	897	**1600.78**	**1600.78**	**1304.52**	24066	1644.88	TL
pr439	439	50	1760.86	899.70	TL	1108	**1350.00**	**1350.00**	**302.98**	4413	1467.35	TL
pr439	439	60	1550.00	753.42	TL	1040	**1150.27**	**1120.79**	TL	19888	1340.01	TL
pr439	439	70	1308.63	707.85	TL	985	**1006.23**	**982.66**	TL	20694	1231.11	1316.50
pr439	439	80	1129.71	651.34	TL	1396	**915.49**	**873.76**	TL	18587	1217.58	955.74
pr439	439	90	1025.91	603.73	TL	1421	**813.94**	**752.26**	TL	17207	986.47	723.38
rat575	575	10	220.06	44.70	TL	242	**138.85**	**138.85**	**19.41**	0	140.52	TL
rat575	575	20	137.20	39.93	TL	300	**93.43**	**93.43**	**705.69**	2335	94.64	TL
rat575	575	30	94.94	36.76	TL	396	**72.09**	**71.31**	TL	8725	74.52	1101.33
rat575	575	40	82.04	34.21	TL	387	66.61	**59.49**	TL	4803	**64.88**	950.51
rat575	575	50	73.00	32.58	TL	490	57.25	**51.90**	TL	6189	**56.94**	717.39
rat575	575	60	63.29	31.91	TL	489	53.74	**46.82**	TL	4661	**51.35**	595.10
rat575	575	70	55.15	30.95	TL	597	**47.42**	**42.44**	TL	7099	47.85	494.17
rat575	575	80	50.96	30.29	TL	309	45.28	**39.05**	TL	7689	**44.29**	448.23
rat575	575	90	48.30	29.73	TL	493	44.69	**36.25**	TL	9866	**41.11**	319.13
rat575	575	100	44.27	29.16	TL	409	40.79	**34.13**	TL	10831	**38.63**	247.96
rat783	783	10	254.92	42.13	TL	0	**163.68**	**163.68**	**54.58**	0	166.23	TL
rat783	783	20	162.75	40.00	TL	46	**109.57**	**109.57**	**841.93**	1016	112.70	TL
rat783	783	30	111.57	37.33	TL	83	**83.55**	**83.49**	TL	3938	88.57	TL
rat783	783	40	97.00	35.01	TL	77	76.90	**70.18**	TL	2296	**76.03**	TL
rat783	783	50	86.58	33.37	TL	67	68.66	**60.76**	TL	2219	**66.10**	TL
rat783	783	60	74.33	32.64	TL	161	61.40	**54.92**	TL	2864	**60.02**	1617.55
rat783	783	70	65.37	31.94	TL	70	59.03	**50.25**	TL	2403	**55.44**	1642.05
rat783	783	80	60.61	31.34	TL	91	56.14	**46.10**	TL	3000	**51.66**	1420.24
rat783	783	90	56.04	30.95	TL	70	50.49	**43.09**	TL	4618	**48.47**	1211.55
rat783	783	100	53.14	30.47	TL	65	47.76	**40.36**	TL	4175	**45.88**	1019.60
pr1002	1002	10	6435.06	1251.44	TL	0	**5202.16**	**5202.16**	**107.49**	0	5331.28	TL
pr1002	1002	20	4606.52	1169.60	TL	0	**3170.57**	**3170.57**	**135.59**	44	3290.14	TL
pr1002	1002	30	3431.11	1073.20	TL	0	**2631.54**	**2502.77**	TL	2470	2644.33	TL
pr1002	1002	40	2983.29	1042.25	TL	0	**2210.20**	**2140.22**	TL	3850	2304.89	TL
pr1002	1002	50	2562.23	996.93	TL	11	2015.56	**1841.90**	TL	3006	**2013.08**	TL
pr1002	1002	60	2241.09	952.64	TL	0	1874.17	**1681.42**	TL	3965	**1838.48**	TL
pr1002	1002	70	2015.56	935.16	TL	15	1732.77	**1507.48**	TL	2889	**1710.26**	TL
pr1002	1002	80	1860.78	899.33	TL	3	1565.25	**1391.70**	TL	3657	**1518.22**	TL
pr1002	1002	90	1718.28	862.03	TL	9	**1431.36**	**1283.52**	TL	5200	1442.22	TL
pr1002	1002	100	1569.24	842.84	TL	9	1414.21	**1208.88**	TL	5803	**1353.70**	TL
rl1323	1323	10	8524.65	1470.07	TL	0	**6229.60**	**6193.80**	TL	0	6313.82	TL
rl1323	1323	20	5699.21	1041.99	TL	0	**3845.66**	**3832.86**	TL	440	4032.83	TL
rl1323	1323	30	3992.96	973.42	TL	0	3906.16	**2984.30**	TL	111	**3204.16**	TL
rl1323	1323	40	3375.04	923.26	TL	0	**2652.14**	**2502.04**	TL	388	2774.72	TL
rl1323	1323	50	2963.63	913.79	TL	0	**2308.32**	**2198.20**	TL	766	2430.27	TL
rl1323	1323	60	2505.42	848.05	TL	0	2495.02	**1947.60**	TL	407	**2149.14**	TL
rl1323	1323	70	2317.57	876.96	TL	0	**1918.35**	**1778.52**	TL	1047	1997.22	TL
rl1323	1323	80	2144.00	863.91	TL	0	1973.72	**1646.13**	TL	1105	**1842.10**	TL
rl1323	1323	90	2025.01	835.69	TL	0	1751.21	**1530.92**	TL	1031	**1745.58**	TL
rl1323	1323	100	1890.05	811.18	TL	0	1624.22	**1429.61**	TL	1185	**1620.92**	TL

The tables show that for 114 out of 154 instances our approaches improve on the best solution value obtained in Sánchez‐Oro et al. [31] and additionally for 7 instances, we match the best solution value. Our approaches manage to solve 76 instances to proven optimality. For some of the instances, our approaches are more than two orders of magnitude faster than the GRASP (e.g., instance pr439 with p=30 and α=2). Comparing 1HSVL with 2HVSL, we can see that 2HVSL performs better overall, in particular for larger instances. This can be explained by the fact that due to the structure of the formulations, the variable fixing procedures can fix much more variables when using (APC2) compared to (APC1). We can also see that for α=3 the problem is harder than for α=2.

In Table 5 we provide a comparison with the local search of  [29]. The runs in [29] were made on an Intel Core i5‐6200 with 2.3 GHz CPU and 8 GB of RAM. We note that  [29] presents runtimes for different version of their developed heuristics, in the table we show the fastest runtime and the best objective function value found by the heuristics. The results show that 2HVSL can solve all instances to optimality under one minute, while for two of the instances the heuristics of  [29] do not manage to find the optimal solution. Similar to the instance set TSPLIB, the setting 1HSVL performs worse than 2HVSL.

**TABLE 5:** Detailed results for instance set pmed with α=2.

			1HSVL				2HVSL				[29]	
Name	|N|	p	UB	LB	t[s]	nBC	UB	LB	t[s]	nBC	UB	t[s]
pmed1	100	5	**150**	**150**	24.16	103	**150**	**150**	**0.34**	0	**150**	0.01
pmed2	100	10	**121**	**121**	20.73	12	**121**	**121**	**0.31**	0	**121**	0.20
pmed3	100	10	**121**	**121**	55.80	325	**121**	**121**	**0.54**	32	**121**	0.26
pmed4	100	20	**97**	**97**	51.19	545	**97**	**97**	**0.91**	345	**97**	8.19
pmed5	100	33	**63**	**63**	4.83	0	**63**	**63**	**0.23**	0	**63**	0.02
pmed6	200	5	**99**	**99**	1009.08	2451	**99**	**99**	**0.37**	0	**99**	0.03
pmed7	200	10	85	74	TL	4207	**80**	**80**	**0.91**	7	**80**	0.09
pmed8	200	20	**70**	66	TL	3839	**70**	**70**	**1.10**	63	**70**	0.03
pmed9	200	40	**49**	**49**	1725.43	3205	**49**	**49**	**0.89**	54	**49**	0.73
pmed10	200	67	**28**	**28**	22.72	7	**28**	**28**	**0.51**	0	**28**	0.62
pmed11	300	5	73	55	TL	1793	**68**	**68**	**0.59**	0	**68**	0.00
pmed12	300	10	72	53	TL	1851	**60**	**60**	**1.16**	0	**60**	0.27
pmed13	300	30	47	41	TL	1760	**43**	**43**	**2.38**	70	**43**	2.07
pmed14	300	60	38	33	TL	3177	**34**	**34**	**2.99**	148	**34**	0.93
pmed15	300	100	24	**23**	TL	4287	**23**	**23**	**1.85**	156	**23**	6.86
pmed16	400	5	56	46	TL	894	**52**	**52**	**0.94**	0	**52**	0.24
pmed17	400	10	56	39	TL	499	**45**	**45**	**3.00**	48	**45**	0.04
pmed18	400	40	44	32	TL	903	**34**	**34**	**4.25**	75	**34**	17.76
pmed19	400	80	29	23	TL	1242	**24**	**24**	**12.88**	836	25	0.17
pmed20	400	133	22	18	TL	1941	**19**	**19**	**3.88**	273	**19**	1.24
pmed21	500	5	59	34	TL	310	**45**	**45**	**1.96**	4	**45**	1.20
pmed22	500	10	52	34	TL	247	**44**	**44**	**4.22**	10	**44**	0.42
pmed23	500	50	36	25	TL	399	**27**	**27**	**7.08**	52	**27**	11.18
pmed24	500	100	23	**19**	TL	511	**19**	**19**	**27.88**	1081	20	0.54
pmed25	500	167	19	**15**	TL	559	**15**	**15**	**15.84**	1501	**15**	33.68
pmed26	600	5	57	35	TL	202	**43**	**43**	**2.21**	0	**43**	0.24
pmed27	600	10	44	29	TL	198	**36**	**36**	**3.42**	0	**36**	0.09
pmed28	600	60	28	21	TL	199	**22**	**22**	**7.46**	30	**22**	0.59
pmed29	600	120	22	16	TL	301	**17**	**17**	**11.25**	78	**17**	0.32
pmed30	600	200	17	**13**	TL	494	**13**	**13**	**11.08**	500	**13**	2.89
pmed31	700	5	47	28	TL	0	**34**	**34**	**3.26**	0	**34**	0.05
pmed32	700	10	46	26	TL	0	**33**	**33**	**5.59**	3	**33**	0.21
pmed33	700	70	24	17	TL	131	**19**	**19**	**13.94**	40	**19**	10.28
pmed34	700	140	18	13	TL	158	**14**	**14**	**54.78**	981	**14**	97.77
pmed35	800	5	43	26	TL	0	**34**	**34**	**4.37**	0	**34**	0.54
pmed36	800	10	49	0	TL	0	**31**	**31**	**9.74**	3	**31**	0.25
pmed37	800	80	24	17	TL	25	**18**	**18**	**35.20**	210	19	0.12
pmed38	900	5	54	23	TL	0	**33**	**33**	**7.89**	0	**33**	0.09
pmed39	900	10	39	21	TL	0	**26**	**26**	**11.40**	13	**26**	0.18
pmed40	900	90	22	14	TL	0	**16**	**16**	**19.91**	44	**16**	3.04

## 7 CONCLUSIONS

In this work, we present two integer programming formulations for the discrete version of the α‐neighbor p‐center problem (d‐α‐pCP), which is an emerging variant of the classical discrete p‐center problem (d‐pCP ), which recently got attention in literature. We also present lifting procedures for inequalities in the formulations, valid inequalities, optimality‐preserving inequalities and variable fixing procedures. We provide theoretical results on the strength of the formulations and convergence results for the lower bounds obtained after applying the lifting procedures or the variable fixing procedures in an iterative fashion. These results extend results obtained by Elloumi et al. [14] and Gaar and Sinnl [16] for the d‐pCP . Based on these results we provide two branch‐and‐cut algorithms, namely one based on each of the two formulations.

We assess the efficacy of our branch‐and‐cut algorithms in a computational study on instances from the literature. The results show that our exact algorithms outperforms existing algorithms for the d‐α‐pCP. These existing algorithms are heuristics, namely a GRASP by Sánchez‐Oro et al. [31] and a local search by Mousavi [29]. Our algorithms manage to solve 116 of 194 instances from literature to proven optimality within a time limit of 1800 seconds, in fact many of them are solved to optimality within 60 seconds. They also provide improved best solution values for 116 instances from literature. Note that these 116 instances are not the same instances as the instances where optimality is proven, as for some of the latter instances the existing heuristics already manage to find the optimal solution (but of course can not prove optimality, as they are heuristics).

There are various directions for further work. One direction could be to try to derive further valid inequalities. In particular it could be interesting to investigate if there are inequalities which ensure that the best possible bounds of both formulations coincide, that is, if the second formulation can be further strengthened, as our current results show that the best bound of the first formulation could be better for some instances. Another interesting avenue could be the development of a projection‐based approach similar to the one of Gaar and Sinnl [16] for the d‐pCP , in which a lower number of variables suffices to model the problem and which is therefore better suited for large scale instances.

Furthermore, trying to extend the approaches including the lifting schemes to other variants of the d‐pCP such as robust versions (see, e.g., [27]), capacitated versions (see, e.g.,  [32]) or the p‐next center problem (see, e.g.,  [26]) could be fruitful. Moreover, while we managed to improve many of the best known solution values for the instances from literature, there are also some instances where the existing heuristic work better. Thus further developments of heuristics can also be interesting, including matheuristics such as local branching (see, e.g., [15]) which could exploit our formulations.

## Data Availability

The data that support the findings of this study are available from the corresponding author upon reasonable request.

## Footnotes

^1^
Unfortunately it is not possible to combine addLocal with purgeable. Thus, outside of the root‐node, the linear programs solved within the B&C tree can contain redundant inequalities.

^2^
Every nonnegative value should already be fine to give higher priority, the documentation is unfortunately not very clear about this, see https://www.ibm.com/docs/en/icos/20.1.0?topic=cm‐setpriority‐method.

## APPENDIX A FORMULATIONS FOR THE d‐pCP FROM THE LITERATURE

### A.1

A formal definition of the d‐pCP is as follows. Given an integer p, a set of customer demand points I with cardinality |I|=n, a set of potential facility locations J of cardinality |J|=m≥p and a distance $d_{ij}$ from a customer demand point i to the potential facility location j for every i∈I and j∈J, find a subset S⊆J with cardinality |S|=p of facilities to *open* such that the maximum distance between a customer demand point and its closest open facility is minimized, that is, such that $\max_{i\in I}\min_{j\in S}\{d_{ij}\}$ is minimized. We note that in the d‐α‐pCP, we have N=I=J by definition of the problem. This is necessary as the set of demand points (i.e., customers) in the d‐α‐pCP depends on a given feasible solution and is defined as all points where no facility is opened in the solution. Due to this difference, slightly modified definitions of D and $D_{i}$ are necessary for the d‐pCP below as compared to the ones of d‐α‐pCP above.

Let the binary variables $y_{j}$ for all j∈J indicate whether a facility is opened at location j. Let the binary variables $x_{ij}$ for all i∈I,j∈J indicate whether the customer i∈I is assigned to the open facility j. Let the continuous variables z measure the distance in the objective function. The classical textbook formulation of the d‐pCP (see e.g., Daskin [13]) is as follows.

(A1a)
$$\begin{array}{lll} \left(\text{PC1}\right) & \;\min z,\; & \end{array}$$

(A1b)
$$\begin{array}{ll} & \text{s.t.}\;\underset{j\in J}{\sum }y_{j}=p, \end{array}$$

(A1c)
$$\begin{array}{ll} & \underset{j\in J}{\sum }x_{ij}=1\;\forall i\in I, \end{array}$$

(A1d)
$$\begin{array}{ll} & x_{ij}\leq y_{j}\;\forall i\in I,\forall j\in J, \end{array}$$

(A1e)
$$\begin{array}{ll} & \underset{j\in J}{\sum }d_{ij}x_{ij}\leq z\;\forall i\in I, \end{array}$$

(A1f)
$$\begin{array}{ll} & x_{ij}\in \{0,1\}\;\;\forall i\in I,\forall j\in J, \end{array}$$

(A1g)
$$\begin{array}{ll} & y_{j}\in \{0,1\}\;\forall j\in J, \end{array}$$

(A1h)
$$\begin{array}{ll} & z\in \mathbb{R}. \end{array}$$

In [14] another formulation was introduced: let $D=\{d_{ij}:i\in I,j\in J\}$ denote the set of all possible distances and let $d_{1}$, …, $d_{K}$ be the values contained in D, so $D=\{d_{1},\ldots ,d_{K}\}$. Furthermore there is a binary variable for each value in D that indicates whether the optimal value of the d‐pCP is less or equal than this value. Towards this end let $u_{k}=0$ if all customers have an open facility with distance at most $d_{k-1}$, otherwise $u_{k}=1$ for all k∈{2,…,K}. Then the formulation reads as follows.

(A2a)
$$\begin{array}{llll} \left(\text{PCE}\right) & \;\min & d_{1}+\overset{K}{\underset{k=2}{\sum }}(d_{k} & -d_{k-1})u_{k}, \end{array}$$

(A2b)
$$\begin{array}{llll} & \text{s.t.} & \underset{j\in J}{\sum }y_{j} & \leq p, \end{array}$$

(A2c)
$$\begin{array}{llll} & \; & \underset{j\in J}{\sum }y_{j} & \geq 1, \end{array}$$

(A2d)
$$\begin{array}{llllll} & & u_{k}+\underset{j:d_{ij}<d_{k}}{\sum }y_{j} & \geq 1 & & \forall i\in I,\forall k\in \{2,\ldots ,K\}, \end{array}$$

(A2e)
$$\begin{array}{llllll} & \; & u_{k} & \in \{0,1\}\;\; & & \forall k\in \{2,\ldots ,K\}, \end{array}$$

(A2f)
$$\begin{array}{llllll} & \; & \;y_{j} & \in \{0,1\}\; & & \forall j\in J. \end{array}$$

Let $D_{i}=\{d_{ij}:j\in J\}\setminus \{d_{1}\}$ for i∈I. The modified variant of (PCE) proposed by Ales and Elloumi [2] is as follows.

(A3a)
$$\begin{array}{llll} \left(\text{PCA}\right) & \min & d_{1}+\overset{K}{\underset{k=2}{\sum }}(d_{k} & -d_{k-1})u_{k}, \end{array}$$

(A3b)
$$\begin{array}{llll} & \text{s.t.}\; & \underset{j\in J}{\sum }y_{j} & \leq p, \end{array}$$

(A3c)
$$\begin{array}{llll} & \; & \underset{j\in J}{\sum }y_{j} & \geq 1, \end{array}$$

(A3d)
$$\begin{array}{llllll} & \; & u_{k-1} & \geq u_{k}\; & & \forall k\in \{3,\ldots ,K\}, \end{array}$$

(A3e)
$$\begin{array}{llllll} & \; & u_{k}+\underset{j\in J:d_{ij}<d_{k}}{\sum }y_{j} & \geq 1\;\; & & \forall i\in I,\forall d_{k}\in D_{i}\cup \{K\}, \end{array}$$

(A3f)
$$\begin{array}{llllll} & \; & u_{k} & \in \{0,1\}\; & & \forall k\in \{2,\ldots ,K\}, \end{array}$$

(A3g)
$$\begin{array}{llllll} & \; & y_{j} & \in \{0,1\}\; & & \forall j\in J. \end{array}$$
